# Establishment of a Nomogram-Based Prognostic Model (LASSO-COX Regression) for Predicting Progression-Free Survival of Primary Non-Small Cell Lung Cancer Patients Treated with Adjuvant Chinese Herbal Medicines Therapy: A Retrospective Study of Case Series

**DOI:** 10.3389/fonc.2022.882278

**Published:** 2022-07-08

**Authors:** Bin Luo, Ming Yang, Zixin Han, Zujun Que, Tianle Luo, Jianhui Tian

**Affiliations:** ^1^Department of Oncology, Shanghai Municipal Hospital of Traditional Chinese Medicine, Shanghai University of Traditional Chinese Medicine, Shanghai, China; ^2^Department of Oncology, Longhua Hospital, Shanghai University of Traditional Chinese Medicine, Shanghai, China; ^3^Department of Good Practice Criterion, Longhua Hospital, Shanghai University of Traditional Chinese Medicine, Shanghai, China; ^4^School of Pharmacy, Jiangxi University of Traditional Chinese Medicine, Nanchang, China; ^5^Cancer Institute of Traditional Chinese Medicine, Shanghai Municipal Hospital of Traditional Chinese Medicine, Shanghai University of Traditional Chinese Medicine, Shanghai, China

**Keywords:** primary non-small cell lung cancer (PNSCLC), Chinese herbal medicines (CHMs), nomogram, progression-free survival (PFS), prognostic model

## Abstract

Nowadays, Jin-Fu-Kang oral liquid (JFK), one of Chinese herbal medicines (CHMs) preparations, has been widely used as an adjuvant therapy for primary non-small cell lung cancer (PNSCLC) patients with the syndrome of deficiency of both Qi and Yin (Qi–Yin deficiency pattern) based on Traditional Chinese Medicine (TCM) theory. However, we found insufficient evidence of how long-term CHM treatment influence PNSCLC patients’ progression-free survival (PFS). Thus, using electronic medical records, we established a nomograph-based prognostic model for predicting PNSCLC patients’ PFS involved with JFK supplementary formulas (JFK-SFs) over 6 months, in order to preliminarily investigate potential predictors highly related to adjuvant CHMs therapies in theoretical epidemiology. In our retrospective study, a series of 197 PNSCLC cases from Long Hua Hospital were enrolled by non-probability sampling and divided into 2 datasets at the ratio of 5:4 by Kennard–Stone algorithm, as a result of 109 in training dataset and 88 in validation dataset. Besides, TNM stage, operation history, sIL-2R, and CA724 were considered as 4 highly correlated predictors for modeling based on LASSO-Cox regression. Additionally, we respectively used training dataset and validation dataset for establishment including internal validation and external validation, and the prediction performance of model was measured by concordance index (C-index), integrated discrimination improvement, and net reclassification indices (NRI). Moreover, we found that the model containing clinical characteristics and bio-features presented the best performance by pairwise comparison. Next, the result of sensitivity analysis proved its stability. Then, for preliminarily examination of its discriminative power, all eligible cases were divided into high-risk or low-risk progression by the cut-off value of 57, in the light of predicted nomogram scores. Ultimately, a completed TRIPOD checklist was used for self-assessment of normativity and integrity in modeling. In conclusion, our model might offer crude probability of uncertainly individualized PFS with long-term CHMs therapy in the real-world setting, which could discern the individuals implicated with worse prognosis from the better ones. Nevertheless, our findings were prone to unmeasured bias caused by confounding factors, owing to retrospective cases series.

## Introduction

Cancer is the second leading cause of death in non-communicable chronic diseases, and lung cancer still makes the maximum contribution to cancer-related mortality worldwide (1–3). With rapid economic development and population aging, newly diagnosed lung cancer cases in China will grow with a rate of 70% at least in the coming 20 years (2, 4). Behind this increasing trend, there are approximately 85% of all diagnosed patients with non-small cell lung cancer (NSCLC). At present, it is generally acknowledged that radical resection is the standard and potentially curative treatment for early-stage NSCLC, including stage I, II, and III (patients satisfying certain operative indication) (5). However, the recurrence and metastasis of NSCLC are regarded as a considerable challenge for post-operation patients’ prognoses, with a 5-year survival rate below 20% (6). Even though several outstanding progresses have been made in cancer therapy, patients’ prognoses still remain uncertain all around the world.

TCM has been developed with a unique system of theories (7), more than thousands of years in clinical practices. CHMs, acupuncture, Tai Chi, etc. under the guidance of TCM theory are widely used in China and accepted by patients internationally (8). CHMs are universally accepted in China for its long history of sole/complementary treatment in various cancers (9). To date, many evidence-based investigations have revealed that CHMs play an important role in reducing adverse drug reactions of chemotherapy and radiotherapy, improving therapeutic efficacy and decreasing the risk of recurrence and metastasis in recent years (10–12). Furthermore, a randomized controlled trial showed that TCM treatment prolonged median survival duration for 0.7 months and significantly improved the 1-year survival rate compared with chemotherapy in advanced NSCLC population (*p = 0.035*; 13). But, during the past decades, few quantitative analyses have focused on what are the odds that long-term CHM-treatment can delay PNSCLC patients’ progression for cancer on the basis of conventional treatment, and how they exert synergistic effect on PNSCLC individuals’ survivability. Therefore, for PNSCLC patients treated with integrative therapy, we need a practical model to investigate latent predictors and to apply given predictors to their PFS prediction by calculating the probability, which not only may explain how those latent CHM-related predictors influence their prognoses, but also might infer few individuals with shorter PFS because of probably high-risk metastasis or reoccurrence.

Nomograms, also named alignment diagram, can transform complex regression equations into visual graphs, which makes the results of predictive models more readable and comprehensible. Recently, it has been frequently used for integrating with multiple predictive variables to display their complex correlation based on multivariate regression analysis, by using line segments with the scale of a certain proportion on the horizontal plane. What’s more, investigators can weight every level of each variable predictors according to its coefficient of regression, and then add up the total score, related to probability of events (such as metastasis or recurrence), to calculate the patient’s predicted value. In the recent years, combined with multivariate logistic regression model (LRM) and multivariate Cox regression, some researchers apply nomogram to quantifying the difference between various clinical characteristics on survival in NSCLC patients by visualizing predicted values to show its corresponding clinical events, such as progression-free survival (PFS) and overall survival (OS) (14, 15). Although nomogram has been proved to be more precise for predicting survival rate among patients with PNSCLC than traditional TNM staging systems (16, 17), it is scarcely applied to measurement of their prognoses with long-term CHM treatment.

Depending on our pre-phase study that Qi–Yin deficiency pattern is clinically principal syndrome of PNSCLC in accordance with TCM pattern identification (18). And Qi-Yin deficiency pattern, an abstract condition of human body with both Qi-deficiency and Yin-deficiency, is identified from individualized symptoms, pulse, and tongue conforming to TCM clinicians’ knowledge (19). As we know, JFK (San-Jiu Pharmaceutical Co., Ltd., China) is mainly targeted at Qi-Yin deficiency pattern of PNSCLC, which exerts anti-tumor effect under integration of disease and syndrome. Despite the lack of accurate statistical data on JFK’s domestic and foreign applications, we estimated that JFK’s annual applications exceeded 19,400 boxesat home and abroad, using its worldwide annual sales amount as well as estimated retail price. Hence, we tried to establish a nomogram-based prognostic model for PNSCLC individuals treated with adjuvant long-term JFK-SFs in a bid to quantifying the predicted probability of their own PFS—regarding the integration of TCM and modern medicine—simulating complex intervention in real-world clinical circumstance. Furthermore, in compliance with undesirable predicted value, our model might help relevant clinicians to notice several latent individuals with high-risk progression. Similarly, we could infer potential beneficiaries timely as a result of their own desirable predicted PFS. Additionally, *via* the nomogram-based modeling, we may investigate latent predictors associated with PNSCLC patients’ prognosis with long-term CHM treatment, which may support our further study that will focus on how individuals’ survivability is affected by adjuvant TCM treatment. Noteworthily, we conducted the study based on real world data from hospital information system (HIS), and aimed to provide a feasibly theoretical epidemiological approach—taking JFK-SFs as an exemplification of long-term CHM treatment—to evaluating PNSCLC patients’ individualized prognosis in the real-world setting.

## Patients and Methods

### Study Design

**Figure 1:**
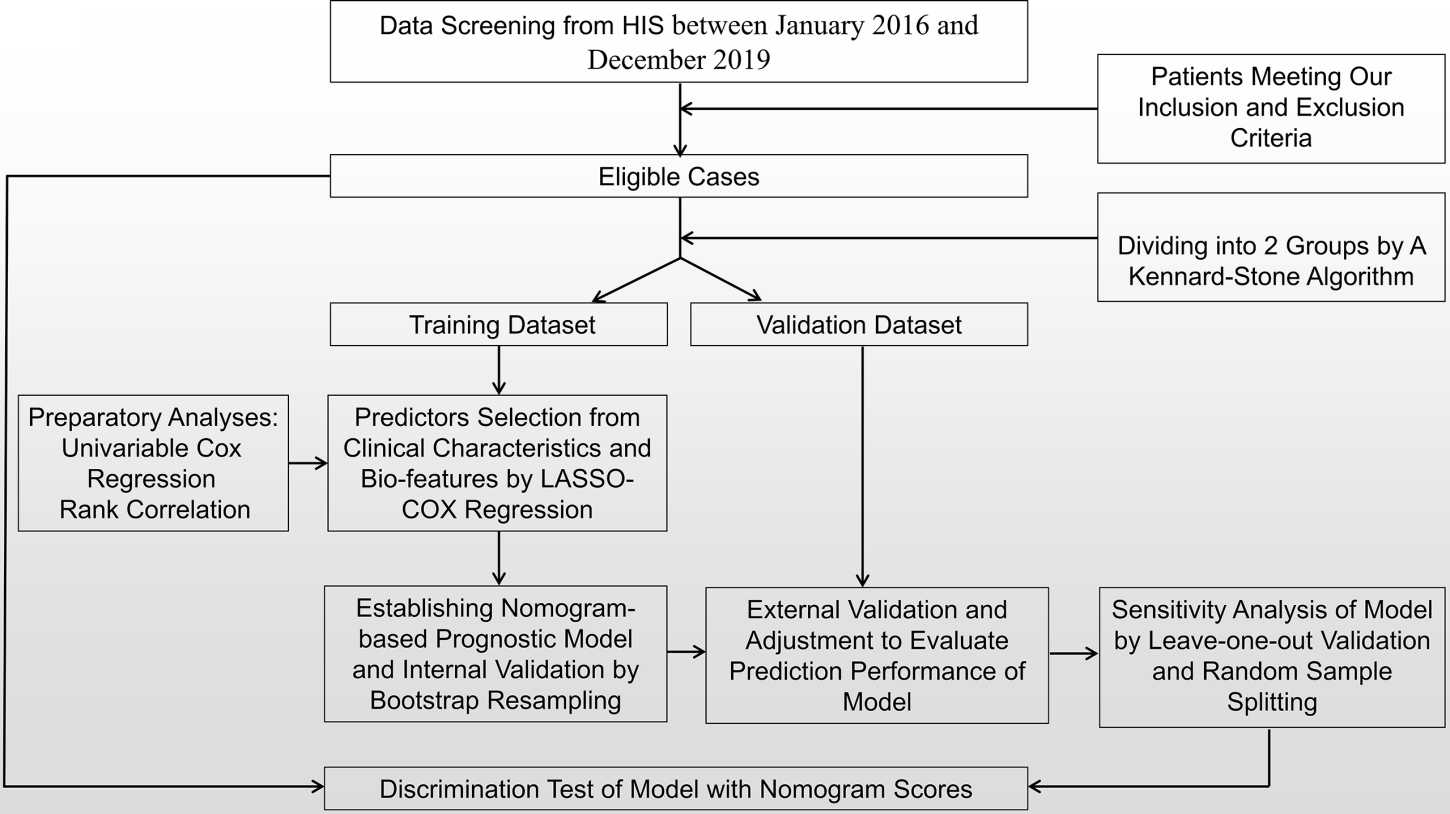
Flowchart of study profile.

Our study protocol was approved by the ethics committee of Long Hua Hospital (2018LCSY022). We performed a retrospective study (between January 2016 and December 2019) of cases series, those who were diagnosed as PNSCLC and accepted long-term adjuvant CHMs decoction, based on HIS from Long Hua Hospital (Class A tertiary hospital), affiliated hospital of Shanghai University of Traditional Chinese Medicine, China. In this investigation, a patient/an individual was seen as a case singly, and the cases matching pre-set inclusion and exclusion criteria were enrolled. In addition, we gained their prognosis information *via* telephone follow-up until December 31, 2022. And we applied the complete data from above-mentioned cases to establishing and modifying a nomogram-based prognostic model, which involved 6 steps in the study procedures (**Figure 1**). Firstly, we screened inpatients with PNSCLC in HIS and selected the eligible cases with our inclusion and exclusion criteria. Secondly, we divided them into training dataset and validation dataset with a certain ratio by Kennard–Stone algorithm, to ensure sufficient samples for modeling and adjustment. Thirdly, combined with multivariable Cox regression analysis, we selected a certain number of prognostic variables that were most significant from clinical characteristics and bio-features based on training dataset by using least absolute shrinkage and selection operator (LASSO), including 2 preparatory analyses: univariable Cox regression and rank correlation presented by heat map. Fourthly, with predictors and calculated PFS, we established prognostic model presented by a nomogram and performed its internal validation *via* bootstrap resampling method. Fifthly, the prognostic model was validated and adjusted based on validation dataset, and we analyzed its prediction performance: discrimination, calibration, and stability (singly examined by sensitivity analysis), with a series of indexes: concordance index (C-index), integrated discrimination improvement (20) and net reclassification indices (NRI). Sixthly, we conducted univariable Cox regression, maximizing Youden’s J statistic, Kaplan–Meier curve and the log-rank test for further examining the discrimination of model with its nomogram scores from all eligible cases. At last, we conducted self-assessment with TRIPOD (Transparent reporting of a multivariable prediction model for individual prognosis or diagnosis) Checklist: Prediction Model Development and Validation.

### Inclusion and Exclusion Criteria

All the inpatients over 18 years who were diagnosed with PNSCLC in pathology and a Qi-Yin deficient pattern based on TCM syndrome identification and had an ECOG score of performance status (Eastern Cooperative Oncology Group) less than or equal to 2 were included. And the exclusion criteria were as follows: (1) cases whose survival duration was shorter than 6 months; (2) cases with other types of malignancies or serious nonmalignant diseases; and (3) cases with incomplete follow-up data; and (4) cases with family history of lung cancer and/or exposure to asbestos.

### Data Extraction and Processing

Judgment sampling, a non-probability sampling method, was used forscreening the eligible PNSCLC cases from HIS. Subsequently, we extracted the data including clinical characteristics and bio-features according to prior knowledge (21, 22). After telephone follow-up to obtain necessary details for calculation of PFS, we covered patients’ information on personal privacy, for example, name and address, to confirm data-processing on condition of anonymity.

### CHMs Therapy

JFK consisting of 12 CHMs can improve PNSCLC patients’ prognoses in some extent, including prolonging survival duration and reducing probability of metastasis, probably viatonifying qi and nourishing yinfor human body, which is consistent with phenomena we observe during clinical practices (23). Notwithstanding its clinically extensive application, under TCM pattern identification—in line with the concept of precision medicine, its supplementary formulas do vary from person to person owing to individual variances in harmony with patients’changing condition on their tongue, pulse diagnoses, other symptoms etc. And detailed information of JFK-SFs with their corresponding TCM syndrome was shown in **Table 1**.We confirmed that all enrolled patients accepted CHMs therapy for at least 6 months from their outpatient and inpatient information records.

**Table 1:** CHM treatment protocol for NSCLC patients with Qi-Yin deficiency pattern.

	Chinese name	Latin name	Dosage (g/per day)
Basic formula	Huang−Qi	*Radix Astragali*	30
	Bei−Sha−Shen	*Radix Glehniae*	30
	Tian−Men−Dong	*Asparagus cochinchinensis*	15
	Nv−Zhen−Zi	*Fructus Ligustri lucidi*	10
	Shi−Shang−Bai	*Herba Selaginellae Doederleinii*	30
	Chong−Lou	*Rhizoma Paridis*	15
	Yin−Yang−Huo	*Herba epimedii*	10
	Jiao−Gu−Lan	*Gynostemma pentaphyllum*	10
	Shan−Zhu−Yu	*Cornus officinalis*	10
	Shi−Jian−Chuan	*Salvia chinensis*	30
	Mai−Dong	*Ophiopogon japonicus*	15
	Hu−Lu−Ba	*Trigonella foenum graecum*	10
Supplementary CHMs for resolving phlegm due to spleen deficiency	Bai−Zhu	*Atractylodes macrocephala*	15
	Ban−Xia	*Rhizoma Pinelliae*	12
Supplementary CHMs for clearing endogenous heat due to disturbance of blood circulation	Dan−Shen	*Radix Salviae Miltiorrhizae*	15
	Dang−Gui	*Radix Angelicae Sinensis*	12

#### Outcome Measurement

On account of little feasibility—that we cannot ensure adequate duration of long-term follow-up—in clinicians’ routine work, we chose 1-year and 2-year PFSas the endpoint in our study to make full use of pre-existing data (24). And PFS, being a commonly used surrogate outcome for prognosis in oncology, was defined as the interval from enrollment date to first documented cancer progression or death of any cause. Nevertheless, if a patient’s date of death could not be retrieved, we applied the last follow-up date to approximate process in our study. To reduce assessment biascaused by assessors, the patients’ informatione xcept for date wash ded during calculating PFS.

### Statistical Analysis

R software (https://www.r-project.org/) was applied to the whole part of analyses in study. We used glmnet package, survival package, RMS package for analyzing LASSO-Cox’s proportional hazards model (LASSO-COX Regression), PFS, creating and modifying nomogram respectively.

#### Statistical Description and Inference

Original data were summarized as mean ± standard deviation (25) or median (interquartile range, IQR), where applicable. What needed to be interpreted was that absolute count of immune cells was described by median (IQR), but was standardized by log-transformed before modeling tomeet theproportional hazards assumption (**Table S1**). We preformed hypothesis testing at the significance level of 0.05 with two-sided test, and *p* as considered as statistical significance.

#### Sampling Error

In this exploratory research, we execute one-off sampling without involvement in parameter estimation.In other words, we had no intention of applying sample statistic (included cases in our study) to inferring population parameter (PNSCLC population with Qi-Yin deficiency who accepted JFK-SFs from HIS). And an estimated confidence interval might be invalid because of lacking repeated sampling for estimating standard error. In summary, population’s features can be inferred from our samples qualitatively rather than quantitatively.

#### Establishment and Validation of Nomogram-Based Prognostic Model

First of all, we divided selected samples into training dataset and validation dataset with the ratio of 5:4 by Kennard–Stone algorithm (25, 26). Besides, we established LASSO-COX regression based on training dataset to select prognostic variables for PFS evaluation with following procedure concretely: (1)optimal value of the penalty parameter corresponding to lambda (λ) in LASSO was chosen by performing leave-one-out cross-validation (LOOCV); (2) the selected lambda (λ) was determined by the smallest LOOCV based on partial-likelihood deviance; (3) those selected variables with non-zero coefficients dependent on their information characteristics by LASSO were used for multivariable Cox regression analysis and the ones with statistical significance (*p* < 0.05) were entered into the nomogram-based prognostic model as predictors finally, which presented predicted results with nomogram; and (4) we performed a bootstrap resampling method, introduced by Ewout Steyerberg (27), for internal validation as well as a primary assessment of predictive power with C-index, NRI, and IDI. Additionally, we conducted external validation based on validation dataset, and used three of the same parameters, for describing its prediction performance after internal validation and establishment with selected predictors. Simultaneously, we assessed its stability by sensitivity analysis (leave-one-out validation and random sample splitting), and discussed predictors’ contribution to PFS prediction by adjusting model parameters. In this procedure, we modified the model with improvement of modeling power presented by IDI > 0 as well as NRI > 0, and we compared the predicted values with the observed ones for modeling calibration of probability of 1-year PFS (1-PFS) and 2-year PFS (2-PFS), which served as a bias correction.

## Results

### Data Screening

From January 2016 to December 2019, there were totally 218 patients with PNSCLC in HIS, and 197 cases among them met both inclusion and exclusion criteria of our study. The reasons for excluded cases that included: 8 patients without documented information of TNM stage and 5 patients withdrawing from the follow-up, 7 patients without records of immune cells, 1 death case owing to postoperative serious complications. And a total of 197 eligible cases were enrolled with the result of 109 in training dataset and 88 invalidation dataset, at the ratio of 5:4 by Kennard–Stone algorithm. The data screening process was entirely displayed in **Figure S1**.

#### General Information of Variables

As to all-round utilization of our clinical data resources, 33 underlying prognostic variables for initial screening were included, such as age, sex, smoking history, TNM stage, pathological types of PNSCLC, treatment protocol (radical resection for lung cancer, mainly platinum-based chemotherapy, radiotherapy, targeted therapy, CHMs therapy), immune cells, cytokines, tumor makers **(** **Tables S2, S3****)**. And 33 abovementioned variables and PFS between training and validation dataset at baseline were summarized in **Table 2**. There were 62 male patients (56.92%) with the mean (25) age of 62.30 (8.92) years in training dataset, while 54 male patients (61.40%) with the mean (25) age of 62.68 (8.98) years invalidation dataset. In terms of advanced patients (TNM stages = IIIb–IV), 25 (22.90%) and 16 (18.20%) were respectively found in training dataset and validation dataset. Compared with 75 (85.20%) of adenocarcinoma (ADC), 9 (10.20%) of squamous cell carcinoma (SCC), and 4 (4.55%) of other types of PNSCLC in validation dataset, the corresponding proportion of training dataset respectively were 89 (81.70%), 16 (14.70%), and 4 (3.60%), from patients’ pathological diagnosis. In addition, our finding showed that the median follow-up time in training dataset was 30.47 months (ranging from 22.27 to 33.27 months) and 30.55 months (ranging from 21.15 to 33.37 months) in validation dataset.

**Table 2:** Baseline data of general information in two datasets.

Items	Training dataset	Validation dataset
No. of patients	109	88
Sex = 2(male) (%)	62 (56.90)	54 (61.40)
Age (mean (SD))	62.30 (8.92)	62.68 (8.98)
PFS (median [IQR])	30.47 [22.27~33.27]	30.55 [21.15~33.37]
TNM Stage 2 = 3b–4 (%)	25 (22.90)	16 (18.20)
Pathological diagnosis (%)		
ADC=1	89 (81.70)	75 (85.22)
SCC=2	16 (14.70)	9 (10.23)
Others=3	4 (3.60)	4 (4.55)
Smoking = no (%)	82 (75.2)	72 (81.8)
Treatment		
Operation = no (%)	25 (22.9)	12 (13.6)
Chemotherapy = no (%)	51 (46.8)	47 (53.4)
Radiotherapy = no (%)	96 (88.1)	78 (88.6)
TT = no (%)	95 (87.2)	79 (89.8)
State, PD = 1 (%)	37 (33.9)	30 (34.1)
PFS (median [IQR])	30.47 [22.27~33.27]	30.55 [21.15~33.37]
Tregs (median [IQR])	4.10[2.64~6.05]	3.64[2.57~6.46]
M-MDSC (median [IQR])	3.47 [2.64~4.73]	3.80 [2.80~4.90]
PMN-MDSC (median [IQR])	18.95 [14.40~25.97]	19.11 [13.61~24.60]
CD3 (median [IQR])	69.50 [65.40~75.10]	68.65 [62.22~75.35]
CD4 (median [IQR])	44.10 [38.30~48.70]	43.00 [38.45~47.90]
CD8 (median [IQR])	23.00 [18.10~26.50]	20.95 [17.00~26.88]
CD4/CD8 (median [IQR])	2.00 [1.48~2.64]	2.10 [1.44~2.62]
CD56 (median [IQR])	14.80[11.00~22.50]	13.50 [10.07~17.98]
CD19 (median [IQR])	12.10[9.20~15.60]	14.45 [9.75~18.32]
IFN-γ (median [IQR])	3.00[1.90~4.90]	4.05 [2.58~7.00]
TGF-β (median [IQR])	208.80 [118.50~284.80]	185.30[119.30~279.92]
TNF-α (median [IQR])	4.70 [3.20~6.10]	4.45 [3.20~5.80]
VEGF (median [IQR])	77.90 [44.30~120.60]	74.75[46.08~117.53]
IL_6 (median [IQR])	2.60 [2.00~4.10]	2.70 [2.00~3.90]
IL_8 (median [IQR])	7.10 [5.00~12.80]	8.25[5.83~12.17]
IL_2 (median [IQR])	36.80 [20.30~72.80]	41.90[22.98~68.80]
sIL_2R (median [IQR])	356.00 [281.00~470.00]	361.50 [271.75~498.00]
CEA (median [IQR])	2.20 [1.40~5.50]	2.45 [1.58~4.50]
AFP (median [IQR])	2.89 [2.19~3.69]	3.05[2.09~4.12]
SCC (median [IQR])	0.90[0.70~1.30]	1.00[0.70~1.22]
CA153 (median [IQR])	11.00 [8.00~16.50]	10.25[7.38~17.10]
CA125 (median [IQR])	15.50 [10.90~24.40]	14.75[9.80~24.10]
CA199 (median [IQR])	10.98 [7.77~15.74]	11.80 [7.38~18.32]
CA724 (median [IQR])	2.63 [1.33~6.06]	2.19[1.21~6.12]
NSE (median [IQR])	12.14 [10.86~13.82]	12.17 [10.72~13.81]
CYFRA211 (median [IQR])	2.44[1.88·3.57]	2.45[1.87~4.05]
SF (median [IQR])	198.50 [103.50~295.10]	183.50[102.93~305.88]

### Predictors’ Selection of Prognostic Model

**Figure 2:**
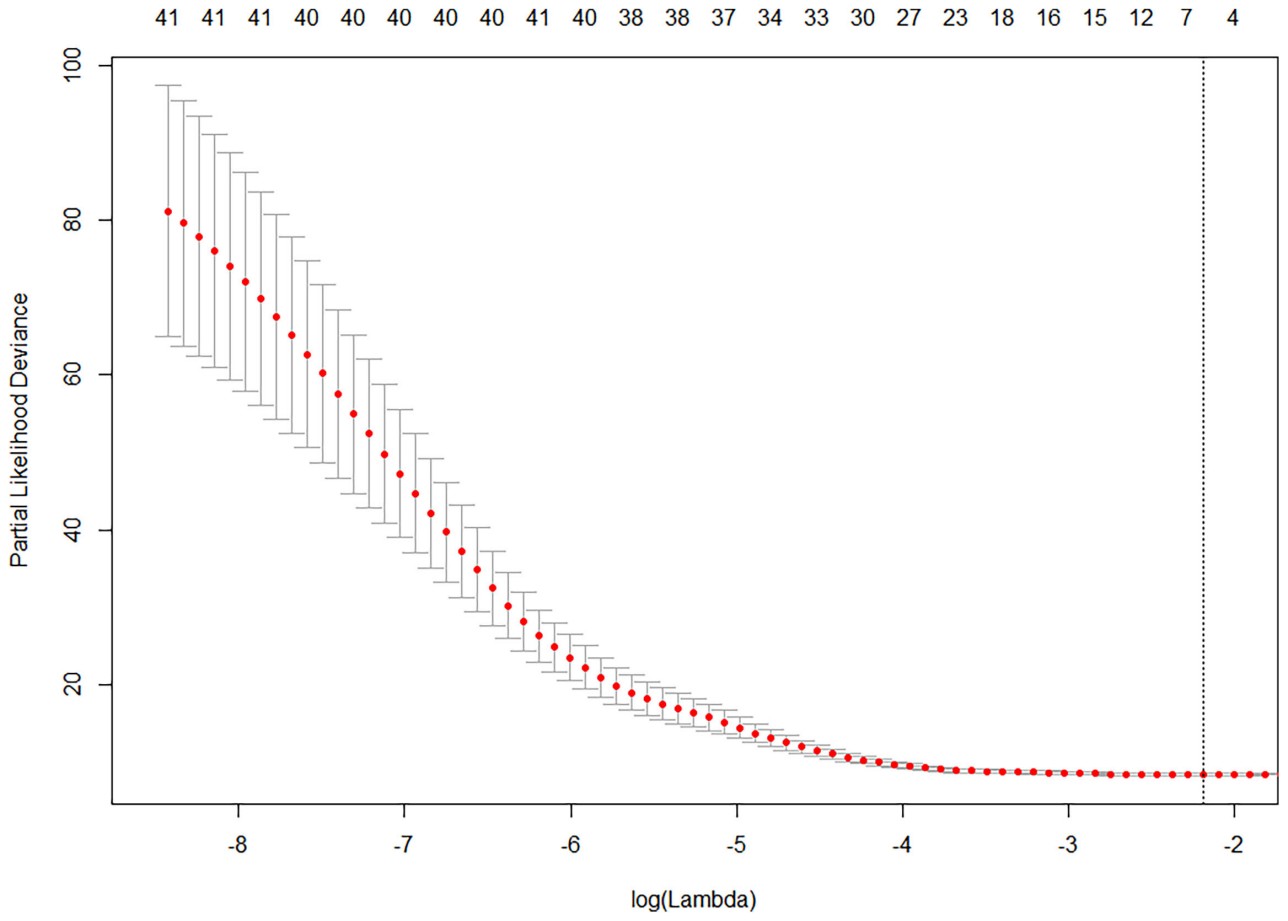
Selecting prognostic variables by using LASSO analysis.

We managed to find predictors (a set of prognostic variables chiefly affecting PFS) by using LASSO-Cox regression for analyzing training dataset (*n* = 109). First, 33 variables from 2 datasets were compared by using univariable Cox regression (**Table 3**), providing a reference for further selection of independent predictors, and we found statistical significance of TNM stage, operation history, chemotherapy, targeted therapy, M-MDSC, CD3, CD56CD16(NK), IL-6, SIL-2R, CEA, CA153, CA152, CA199, CA724, NSE, and CYFRA211 in both datasets, which suggested that they might be implicated predictors for predicting PFS. Second, we used Spearman’s correlation coefficient (12) for evaluating negative or positive correlation/non-correlation between bio-features and clinical characteristics in pairs (**Figure S2**). Third, a total of 33 selected variables were entered into the LASSO model. As shown in **Figure 2**, the optimal log(lambda) was achieved at the value of -2.1895 (lambda = 0.112) by the minimum LOOCV based on partial-likelihood deviance, generating reduction of variables and attaining 7 prognostic variables: TNM stage, operation history, targeted therapy (TT), IL-6, sIL-2R, CA153, and CA724. And according to the result of weighted Schoenfeld residuals test, the 7 variables were qualified for proportional hazards assumption (*p* > 0.05). Fourth, we gained 4 of 7 variables because of their statistical significance (*p* < 0.05) by multivariant Cox regression analysis. In brief, TNM stage, operation history, sIL-2R (immune cytokines), and CA724 (tumor marker) were considered as predictors for predicting individuals’ PFS, which would be put into nomogram-based prognostic model (**Table S4**).

**Table 3:** Comparisons between two datasets by the univariable Cox regression analysis.

Classification	Variables	Training dataset			Validation dataset		
		HR	95% CI	*p* value	HR	95% CI	*p* value
Clinical characteristics	Sex						
	Female (28)	Reference					
	Male (1)	0.582	0.305~1.112	0.101	0.345	0.166~0.719	0.000
	Age	1.018	0.982~1.057	0.331	1.070	1.027~1.115	0.000
	Smoking						
	Yes	Reference					
	No	0.537	0.273~1.055	0.071	0.420	0.192~0.921	0.030
	TNM stage						
	Stage 1 (1–3a)	Reference					
	Stage 2 (3b–4)	13.257	6.582~26.701	0.000	9.313	4.476~19.376	0.000
	Pathological diagnosis						
	ADC	Reference					
	SCC	1.163	0.483~2.801	0.737	2.694	1.018~7.128	0.050
	Others	1.717	0.409~7.200	0.460	3.872	1.151~13.019	0.030
	Treatment						
	Operation						
	Yes	Reference					
	No	8.22	4.223~16.004	0.000	6.137	2.806~13.422	0.000
	Chemotherapy						
	Yes	Reference					
	No	0.201	0.088~0.459	0.000	0.244	0.108~0.548	0.000
	Radiotherapy						
	Yes	Reference					
	No	0.657	0.274~1.578	0.348	0.493	0.188~1.292	0.150
	Targeted therapy						
	Yes	Reference					
	No	0.127	0.063~0.256	0.000	0.126	0.057~0.282	0.000
Bio-features	Tregs	1.049	0.969~1.136	0.237	1.023	0.922~1.136	0.670
	M-MDSC	1.223	1.050~1.424	0.010	1.388	1.162~1.657	0.000
	PMN-MDSC	1.012	0.975~1.052	0.528	1.026	0.983~1.07	0.240
	CD3	0.964	0.935~0.994	0.018	0.953	0.918~0.989	0.010
	CD4	0.982	0.949~1.016	0.303	0.952	0.914~0.99	0.010
	CD8	0.968	0.923~1.015	0.181	0.985	0.938~1.035	0.550
	CD4/CD8	1.086	0.778~1.514	0.628	0.885	0.588~1.33	0.560
	CD56/CD16(NK)	1.04	1.011~1.069	0.006	1.053	1.019~1.088	0.000
	CD19	0.953	0.886~1.025	0.199	0.975	0.914~1.04	0.440
	LogCD3	0.122	0.048~0.310	0.000	0.035	0.005~0.234	0.000
	LogCD4	0.152	0.035~0.662	0.012	0.059	0.012~0.3	0.000
	LogCD8	0.122	0.028~0.528	0.005	0.115	0.022~0.608	0.010
	LogCD56CD16NK	1.552	0.451~5.344	0.486	1.196	0.232~6.16	0.830
	LogCD19	0.268	0.089~0.805	0.019	0.206	0.066~0.643	0.010
	IFN	0.986	0.928~1.047	0.642	0.966	0.894~1.044	0.390
	TGF2	1.002	0.999~1.004	0.221	1.002	0.999~1.005	0.210
	TNF1	1.004	0.982~1.026	0.745	1.007	0.972~1.043	0.710
	VEGF	1.001	0.998~1.004	0.411	1.000	0.994~1.005	0.870
	IL-6	1.103	1.057~1.152	0.000	1.101	1.044~1.161	0.000
	IL-8	1.027	0.998~1.056	0.068	1.043	0.978~1.112	0.200
	IL-2	0.999	0.995~1.003	0.559	1.000	0.997~1.004	0.920
	sIL-2R	1.003	1.002~1.004	0.000	1.005	1.003~1.007	0.000
	CEA	1.004	1.002~1.005	0.000	1.007	1.002~1.012	0.010
	AFP	1.045	0.822~1.328	0.722	1.040	0.856~1.264	0.690
	SCC	1.199	0.944~1.524	0.137	1.226	1.034~1.455	0.020
	CA153	1.069	1.036~1.103	0.000	1.028	1.009~1.047	0.000
	CA125	1.006	1.003~1.008	0.000	1.006	1.003~1.009	0.000
	CA199	1.002	1.001~1.004	0.009	1.003	1.001~1.006	0.020
	CA724	1.047	1.024~1.071	0.000	1.040	0.993~1.09	0.100
	NSE	1.161	1.070~1.260	0.000	1.116	1.008~1.237	0.040
	CYFRA211	1.023	1.010~1.037	0.001	1.046	1.024~1.069	0.000
	SF	1.002	1.001~1.003	0.002	1.000	0.999~1.002	0.610

### Establishment and Internal Validation of Prognostic Model

**Figure 3:**
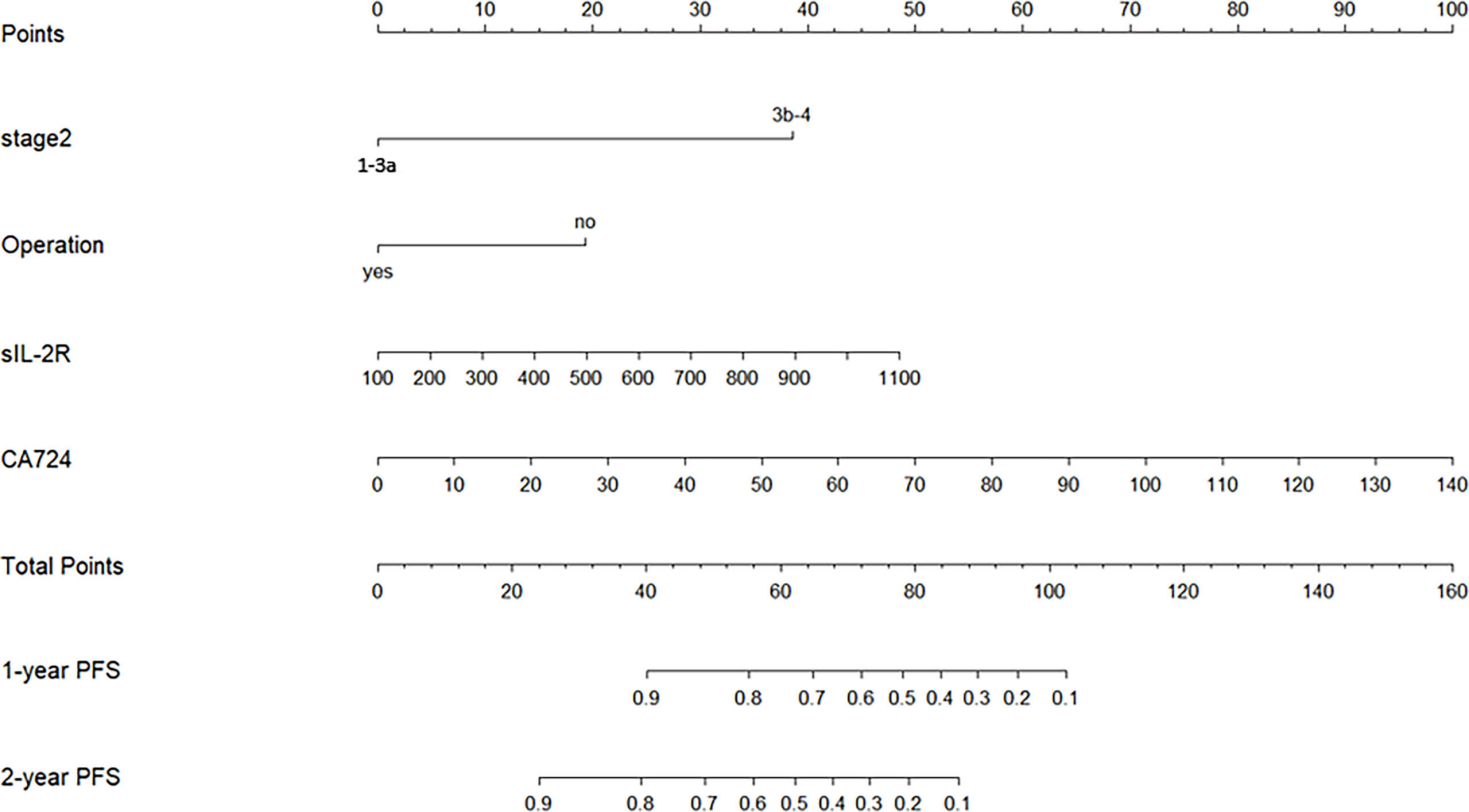
Nomogram-based prognostic model for NSCLC patients of Qi-Yin deficiency pattern with long-term CHM treatment (*Stage 2*, TNM stage; *Operation*, operation history; *1-year PFS*, predicted probability of PFS of 1 year; *2-year PFS*, predicted probability of PFS of 2 years).

As it was shown in **Figure 3**, our nomogram-based prognostic model could graphically display predicted 1-PFS and 2-PFS by incorporating 4 prognostic variables (TNM stage, operation history, sIL-2R, and CA724). Each subtype within category characteristics was assigned a score on the line segment with scale of a certain proportion, where each variable was drawn on, for the purpose of describing integrated correlation of PFS probability with them. Eventually, the precisely estimated 1-PFS and 2-PFS were quantified by the percentage transformed from a total accumulated score.

Internal validation was performed by a bootstrap resampling method, whose procedure was repeated 10,000 times for resampling in training dataset, and the decrease from different performance between bootstrap and original sample (training dataset) became the scale for our preliminary estimation of its power. The result suggested that our model was well-calibrated with C-index of 0.836 and bias-corrected C index of 0.829 (**Table S5**). In order to assess the 4 prognostic factors’ degree of contribution to modeling, the following 3 models were taken into consideration as a further calibration: model 1 was the final prognostic model based on the 4 predictors as a baseline model; model 2 was simply established based on 2 clinical characteristics (TNM stage and operation history); model 3 was established in terms of 2 bio-features (sIL-2R and CA724). Then pairwise comparison of C-index and NRI and IDI were made in groups: model 2 vs. model 1 and model 3 vs. model 1, whose additional discriminative power caused by additional predictors compared with a baseline model could represent the better prediction performance. As a result, we found acceptable performance in all of the 3 models and model 1 ranked first with the highest C-index of 0.836 (95% CI: 0.765~0.907), compared to model 2 (0.806, 95% CI: 0.734~0.877) and model 3 (0.702, 95% CI: 0.612~0.796). Next, we calibrated 1-PFS and 2-PFS of models in sequence (**Figure S3**) and also found that model 1 presented the best performance according to the result of pairwise comparison (**Table S6**), in which model 1 provided more evident improvements (IDI > 0 and NRI > 0) in prediction than model 2 and model 3, implying that clinical characteristics and bio-features jointly contributed to improving prediction performance of our prognostic model.

### External Validation and Modification of Prognostic Model

**Figure 4:**
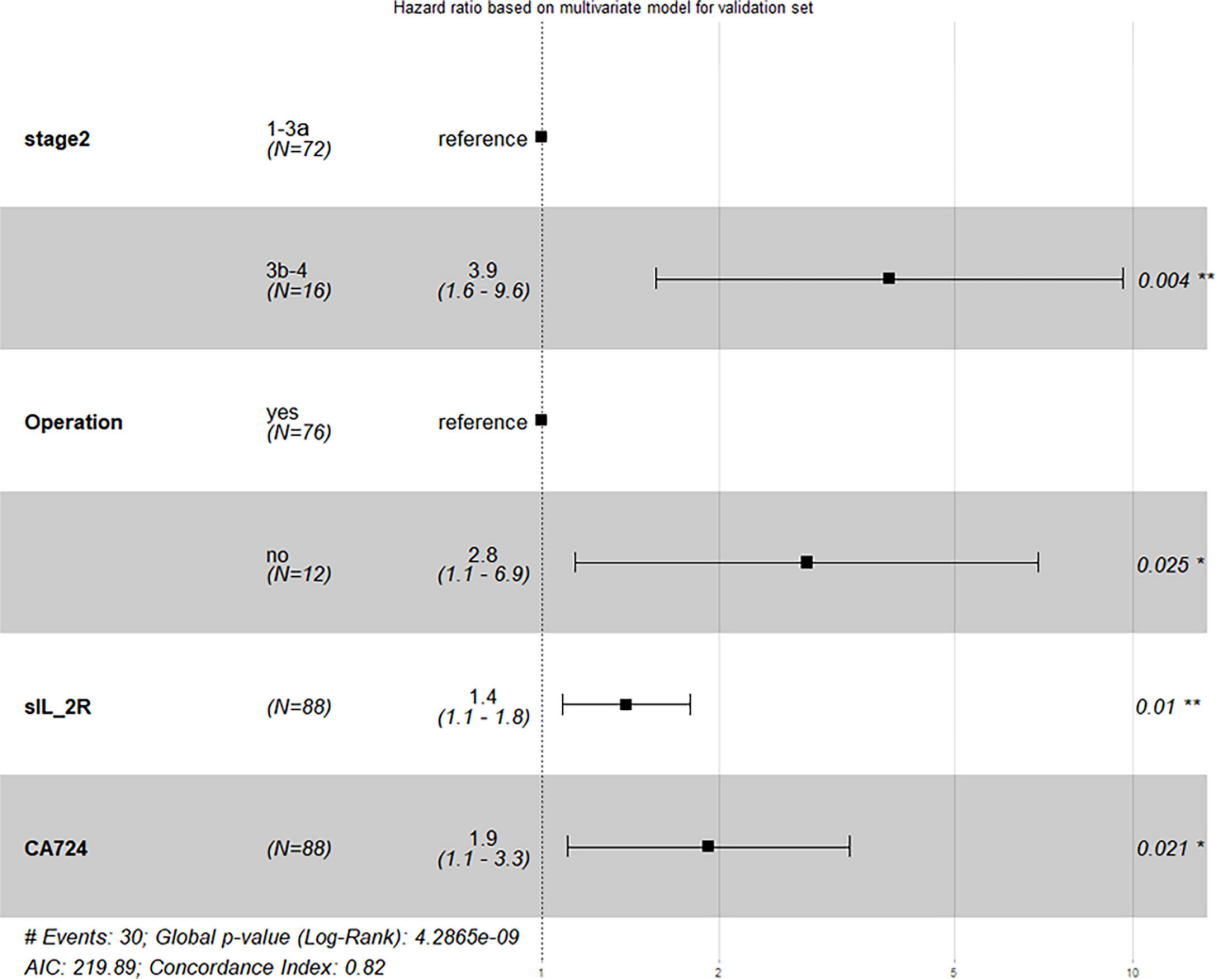
Result of external validation based on validation dataset (*Stage 2*, TNM stage; *Operation*, operation history).

In our study, validation dataset (*n* = 88) was used as an independent set for external validation, which tested predictive power of model. With the same procedures of treating training dataset (*n* = 109), 3 models were established: model 1 with 4 predictors, model 2 with 2 predictors, and model 3 with the others. On this situation, the C-index of model 1, model 2, and model 3 was 0.816 (95% CI: 0.743~0.891), 0.756 (95% CI: 0.679~0.836), and 0.730 (95% CI: 0.649~0.824), respectively. We also modified their 1-PFS and 2-PFS one by one (**Figure S4**), and unobvious distinctions between predicted values and actually observed values in both 1-PFS and 2-PFS were found. In short, model 1 still showed the best performance because all of the 4 predictors met significance level at *p* < 0.05, with C-index of 0.816 (95% CI: 0.743~0.891) based on validation dataset, close to that of the training dataset (**Figure 4**). Similarly, we made pairwise comparison of C-index and NRI and IDI among 3 models (**Table S5**), observing the consistency of that model 1 showed optimal performance.

### Sensitivity Analysis of Prognostic Model

Our model was modified by changing the modeling samples from training dataset (*n* = 109) in a bid to assess its stability. In this part, 2 strategies were applied (28): leave-one-out validation and (1) random sample splitting. For the former (28), as a result of re-modeling based on remaining patients’ data, we left a patient out of the training dataset at a time. Both predicted result and performance of the rest samples were recorded. And we stopped this procedure until every sample from training dataset was excluded once in turn. The distribution of Hazard Ratio (29) values for 4 prognostic factors were shown in **Figure S5A** and their average HR respectively were:6.757 for TNM Stage, 2.600 for operation history, 1.271 for sIL-2R, and1.422 for CA724, with the average C-index of 0.836 (**Figure S5B**). For the latter (1), the overall training dataset(n=109) was randomly divided into modified training dataset (n=61) and modified validation dataset (*n* = 48) based on the same proportion (5:4). And the distribution of HR values and C-index for both modified datasets were shown in **Figures S5C, D**, while HR mean and C-index mean were described in **Table S7** that the average C-index of modified training dataset and validation dataset respectivelywere 0.827 (95%CI:0.782~0.877) and 0.828 (95%CI:0.771~0.886). In a word, all results demonstrated the stability of our prognostic model with 4 predictors, containing 2 bio-features and 2 clinical characteristics.

### Discrimination Testof Prognostic Model

To further examine the discrimination of our nomogram-based prognostic model with its nomogram score, univariable Cox Regression was performed again. As a result, our model respectively yielded HR of 1.053 (95% CI: 1.038~1.068; *p* < 0.001) and 1.037 (95% CI: 1.030~1.062; *p* < 0.001) for training dataset (*n* = 109) and validation dataset (*n* = 88), and a cut-off value of 57 was determined by maximizing Youden’s *J* statistic. Subsequently, patients in both datasets (*n* = 197) were divided into 2 groups: high-risk progression (nomogram score >57) and low-risk progression (nomogram score ≤57). In final, we also statistically tested the significant difference between two groups mentioned above by Kaplan–Meier curve and Log-rank test (*p* < 0.001), which may preliminarily be another feasible application of the model (**Figures S6, S7**). At the end of this manuscript, we finished and submitted a checklist of TRIPOD in **Table S8**.

## Discussion

As is known to us, the integration of TCM and modern medicine became a comprehensive treatment for PNSCLC in China for decades, especially for advanced patients. And long-term CHM treatment, as a common adjuvant therapy for lung cancer, has proven its strengths for prolonging survival duration, controlling metastasis, and reducing mortality (11, 30). However, it is still difficult to explain how modern medicine incorporating TCM interactively respond to individualized prognosis in oncology, not to mention whether adjuvant long-term CHM treatment can prolong individualized survivability for PNSCLC patients. Consequently, combined with our experience from clinical practice, we established a nomogram-based prognostic model of their PFS prediction, by using HIS data from PNSCLC patients who accepted JFK-SFs over 6 months, to explore potential predictors accounting for multiple-layer and multi-dimensional causal effect of integrative treatment. In this retrospective study, we selected 4 predictors (TNM stage, operation history, CA724, and sIL-2R) of 33 available variables for modeling by using LASSO-Cox regression for the reason that LASSO (a data analysis method) is suitable for limiting the amount of variables selection in high dimensional data from limited samples, for example, biomarker selection, which originally proposed for avoidance of overfitting (31, 32). And Song et al. team applied LASSO to establishing prognostic model for predicting personalized PFS of PNSCLC patients with EGFR tyrosine kinase inhibitors therapy (33). Whereas, a sequence of restrictions hindering LASSO from more frequently precise modeling may not be ignored: (1) with achieving parsimony towards vital variables’ coefficients, the result of LASSO regression is undoubtedly biased estimates due to constraint parameter entered (34, 35); (2) without more prior knowledge about their structural sparsity, it seems reasonable that every variable’s coefficient has equal chance of exact shrinkage of all to zero, but a variable with an accurate zero is unlikely to occur in actually most cases (36); (3) despite achieving parsimony, seriously speaking, LASSO is not good at addressing variables with multi-label classification and multi-collinearity, whose coexisting or unexclusive property of interaction for prediction is outside the scope of its typical features’ selection (37). Interestingly, some researches focused on sIL-2R and CA724 that could provide several clues to our further study: (1) sIL-2R attached relatively significance to the tumor immune network, regarded as a possible biomarker for the early detection and follow-up of Nivolumab-induced pneumonitis (38), and high concentration of sIL-2R contributed to the disorder in human’s internal environment that can promote tumorigenesis (39, 40); (2) CA724, a valuable marker for gastric cancer, could be a biomarker for tumor detection of advanced lung cancer (41), and Chen et al. found it was associated with TNM stage of PNSCLC as well as metastasis of lung adenocarcinoma (42), suggesting that its clinical value in PNSCLC prognosis should be laid greater emphasis on. After establishment of model, we aimed to use nomogram for displaying the predicted probability of PFS because it was more precise than TNM staging systems for prognosis prediction (16, 43). Besides, we performed external validation to avoid modeling overfitting and to determine its generalizability (29). In recent years, Zhang et al. established a nomogram-based model that could predict the probability of 3-year and 5-year brain metastases and identify high-risk resected NSCLC populations (44).

Our study is in need of a serious and an objective interpretation because of a couple of limitations and strengths. On one hand, it is the first study of establishing a nomogram-based prognostic model for PNSCLC patients treated with long-term CHMs, which could detect individuals at risk of metastasis or reoccurrence and possible beneficiaries from CHM treatment to a certain degree. And it considered both bio-features and clinical characteristics as predictors for predicting PNSCLC patients’ PFS with integrative treatment, corresponding to a connection between mechanism researches and clinical experiments—which may reveal an anti-cancer effect of CHMs (45–47). More importantly, in the future, we want to predict individualization prognosis involved in TCM individualized therapy for evidence-based clinical decision, based on this preparatory work. On the other, our model was established and validated based on a relatively small number of sample set, only containing 197 cases from single-center HIS, resulting in our model’s potential uncertainties of large-scale application for external PNSCLC patients with similar therapy. But negligently, our study imposed constrains on familial-hereditary and asbestos-exposed individuals that these omitted rare risk factors also put weight on PNSCLC prognosis. Moreover, there were inadequate cases, as another test dataset, to support external testing in further, since we could obtain finite PNSCLC cases with long-term CHM treatment from the existing database. In spite of uncontrolled bias caused by small sample size based on case series that can demonstrate no causal inferences (48) from this work, we intended to flexibly improve and modify the model with collecting an increasing number of eligible data. In addition, we can only identify patients’ medication from prescription of HIS instead of their actually daily drug use, the same as those numerous retrospective studies suffered. Lastly, our study simply e valuated clinician-reported outcomes (CROs) forPNSCLC prognosis, but health-related quality of life (HRQoL) that can trustworthily and accurately reflect benefit from cancer therapies was absent from prolonging PFS assessment. And we will concern ourselves with applying HRQoL instruments, for example Lung Cancer Symptom Scale (49), for PFS assessment of PNSCLC patients with long-term CHMs therapy in further prospective study with a controlled group of non-CHM treatment, if appropriate, in order to promote shared decision-making of clinicians and patients.

## Conclusions

In conclusion, a nomogram-based prognostic model for predicting PFS of PNSCLC patients with long-term CHM treatment was established, which provides references for quantifying PNSCLC patients’ unknown PFS in the comprehensive therapy as well as further verification of TCM-intervened-related predictors. And we can also preliminarily use it for discerning high-risk individuals of PNSCLC progression, from those who accept conventional and TCM treatment in real-world settings. Presumably, individuals with higher nomogram scores (>57) seem to be paid close attention to early screening for metastasis and recurrence. Strictly speaking, confounding as residual factors leading to bias of real-world studies are, we must cautiously interpret our findings in this work.

## Data Availability Statement

The raw data supporting the conclusions of this article will be made available by the authors, without undue reservation.

## Ethics Statement

The studies involving human participants were reviewed and approved by the ethics committee of LongHua Hospital (2018LCSY022). The patients/participants provided their written informed consent to participate in this study. Written informed consent was obtained from the individual(s) for the publication of any potentially identifiable images or data included in this article.

## Author Contributions

JT, BL, and MY conceived the study, analyzed the data, and wrote the paper. ZQ and TL collected and extracted the first-hand data and provided analyses for the datasets. MY conducted statistical analyses. ZH offered suggestions on methodology, modified statistical terminology and polished this manuscript. All authors edited the manuscript and approved of the final version.

## Funding

This project is partly sponsored by Shanghai Sailing Program (20YF1449900 to BL), a Municipal Human Resources Development Program for Outstanding Leaders in Medical Disciplines (2017BR044 to JT), National Natural Science Foundation of China (82174245 to JT, 82174017 to ZQ, and 82104943 to BL), and AiJian Program from Long Hua Hospital (AJ071 to BL).

## Conflict of Interest

The authors declare that the research was conducted in the absence of any commercial or financial relationships that could be construed as a potential conflict of interest.

## Publisher’s Note

All claims expressed in this article are solely those of the authors and do not necessarily represent those of their affiliated organizations, or those of the publisher, the editors and the reviewers. Any product that may be evaluated in this article, or claim that may be made by its manufacturer, is not guaranteed or endorsed by the publisher.
