# Supplementary material for: Establishment of a Nomogram-Based Prognostic Model (LASSO-COX Regression) for Predicting Progression-Free Survival of Primary Non-Small Cell Lung Cancer Patients Treated with Adjuvant Chinese Herbal Medicines Therapy: A Retrospective Study of Case Series
Source: Front Oncol. 2022 Jul 8;12:882278. doi: 10.3389/fonc.2022.882278 (PMC9304868; doi:10.3389/fonc.2022.882278)
Supplement: Supplementary file 1 [file DataSheet_1.doc]

# Supplementary Material

## Supplementary Tables: Tab. S1 to Tab. S6

## Tab.S1 Absolute Count of Immune Cells Standardized by Log-Transformed

| Variables | Training Dataset  （n=109） | Validation Dataset  （n=88） | Eligible Cases  （n=197） |
| --- | --- | --- | --- |
| LogCD3 (median [IQR]) | 2.99 [2.86~3.11] | 3.01 [2.92~3.11] | 3.00 [2.89, 3.11] |
| LogCD4 (median [IQR]) | 2.81 [2.67~2.92] | 2.81 [2.72~2.91] | 2.81 [2.68, 2.91] |
| LogCD8 (median [IQR]) | 2.48 [2.37~2.63] | 2.49 [2.38~2.62] | 2.48 [2.37, 2.63] |
| LogCD16Nk (mean (SD)) | 2.33 (0.26) | 2.30 (0.24) | 2.32 (0.25) |
| LogCD19 (median [IQR]) | 2.25 [2.05~2.37] | 2.33 [2.16~2.47] | 2.26 [2.11, 2.42] |

### Tab.S2 Clinical Characteristics of Eligible Cases (n=197)

| No. | Characteristics | Number of Patients (%) | Percentage |
| --- | --- | --- | --- |
| 1 | Sex |  |  |
|  | Male | 116(58.9) | 58.9 |
|  | Female | 81(41.1) | 41.1 |
| 2 | Age |  |  |
|  | <60 | 45(22.8) | 22.8 |
|  | 60-70 | 136(69.0) | 69.0 |
|  | >70 | 16(8.2) | 8.2 |
| 3 | TNM Stage |  |  |
|  | Stage1(1-3a) | 156(79.2) | 79.2 |
|  | Stage2(3b-4) | 41(20.8) | 20.8 |
| 4 | Pathology |  |  |
|  | Adenocarcinoma, ADC | 164(83.2) | 83.2 |
|  | Squamous cell carcinomas, SCC | 25(12.7) | 12.7 |
|  | Other types of NSCLC | 8(4.1) | 4.1 |
| 5 | Smoking |  |  |
|  | Yes | 43(21.8) | 21.8 |
|  | No | 154(78.2) | 78.2 |
| 6 | Treatment |  |  |
|  | Operation | 160(81.2) | 81.2 |
|  | Chemotherapy | 99(50.3) | 50.3 |
|  | Radiotherapy | 23(11.7) | 11.7 |
|  | Targeted Therapy, TT | 23(11.7) | 11.7 |
|  | Chinese Herbal Medicine, CHM | 197(100) | 100 |
| 7 | Progression of NSCLC |  |  |
|  | Yes | 67(34.0) | 34.0 |
|  | No | 130(66.0) | 66.0 |

### Tab.S3 Bio-features of Eligible Cases (n=197)

| No. | Features | Median [IQR] |
| --- | --- | --- |
| 1 | Tregs | 4.09 [2.58, 6.13] |
| 2 | M-MDSC | 3.68 [2.73, 4.83] |
| 3 | PMN-MDSC | 18.98 [14.02, 25.80] |
| 4 | CD3 | 69.30 [63.40, 75.20] |
| 5 | CD4 | 43.20 [38.30, 48.50] |
| 6 | CD8 | 21.70 [17.50, 26.60] |
| 7 | CD4/CD8 | 2.01 [1.45, 2.63] |
| 8 | CD56CD16 NK | 14.20 [10.50, 20.90] |
| 9 | CD19 | 13.00 [9.40, 17.00] |
| 10 | IFN-γ | 3.40 [2.10, 5.70] |
| 11 | TGF-β | 200.50 [118.70, 283.80] |
| 12 | TNF-α | 4.60 [3.20, 6.00] |
| 13 | VEGF | 77.20 [45.70, 120.60] |
| 14 | IL-6 | 2.60 [2.00, 3.90] |
| 15 | IL-8 | 7.70 [5.10, 12.50] |
| 16 | IL-2 | 40.40 [21.50, 72.20] |
| 17 | sIL-2R | 356.00 [274.00, 476.00] |
| 18 | CEA | 2.30 [1.50, 4.90] |
| 19 | AFP | 2.95 [2.17, 3.86] |
| 20 | SCC | 0.90 [0.70, 1.30] |
| 21 | CA153 | 10.60 [7.50, 16.50] |
| 22 | CA125 | 15.20 [10.30, 24.40] |
| 23 | CA199 | 11.48 [7.54, 17.48] |
| 24 | CA724 | 2.38 [1.27, 6.09] |
| 25 | NSE | 12.16 [10.77, 13.82] |
| 26 | CYFRA211 | 2.44 [1.87, 3.82] |
| 27 | SF | 190.00 [103.50, 298.20] |

IQR: Interquartile range;

1.Tregs: Regulatory T cell;

2.M-MDSC: Monocyte Myeloid-derived suppressor cells;

3.PMN-MDSC: Polymorphonuclear Myeloid-derived suppressor cells;

4-9. CD: Cluster of differentiation; NK: Nature killer cell;

10.IFN-γ: Interferon-γ;

11.TGF-β: Tumor growth factor β;

12.TNF-α: Tumor necrosis factor α;

13.VEGF: Vascular endothelial growth factor;

14.IL-6: Interleukin 6;

15.IL-8: Interleukin 8;

16.IL-2: Interleukin 2;

17.sIL-2R: Soluble interleukin 2 receptor;

18.CEA: Carcinoembryonic antigen;

19.AFP: Alpha fetoprotein;

20.SCC: Squamous cell carcinoma antigen;

21-24.CA: Carbohydrate antigen;

25. NSE: Neuron specific enolase;

26.CYFRA21-1: Soluble fragments of cytokeratin 19;

27.SF: Serum ferritin;

### Tab.S4 Final Predictors of Modeling by LASSO-Cox Regression

| Variable | HR | 95% CI | P -Value |
| --- | --- | --- | --- |
| TNM Stage | 6.7492 | 2.836~16.059 | <0.0001 |
| Operation History | 2.5934 | 1.157~5.813 | 0.02067 |
| sIL-2R | 1.0024 | 1.001~1.004 | 0.00611 |
| CA724 | 1.0359 | 1.013~1.060 | 0.00220 |

HR: Hazard Ratio;

CI: Confidence Interval;

### Tab.S5 Result of Internal Validation by Bootstrap Resampling (Repeating 10000 Times )

| Parameters ^a^. | Original Value ^b^. | Training Value ^c^. | Test Value ^d^. | *P*-Value | Bias-corrected Value |
| --- | --- | --- | --- | --- | --- |
| C-index | 0.8355 | 0.8387 | 0.8324 | 0.0063 | 0.8292 |
| S | 1.0000 | 1.0000 | 0.9399 | 0.0601 | 0.9399 |
| D | 0.2194 | 0.2306 | 0.2094 | 0.0212 | 0.1982 |
| U | -0.0060 | -0.0062 | 0.0061 | -0.0123 | 0.0063 |
| Q | 0.2255 | 0.2368 | 0.2033 | 0.0336 | 0.1919 |

1. S: Calibration Slope; D: Index of Discrimination; U: Unreliability; Q: Overall Quality;
2. Calculation of Training Dataset (n=109);
3. Calculation of Resampling-based Training Data;
4. Calculation of Resampling-based Testing Data;

### Tab.S6 Difference of Prediction Performance Based on Pairwise Comparisons in Models

| Comparisons | Parameters ^a^. | Dataset ^b^. | Predicted Value | Estimate | 95% CI^C^. | | *P*-value |
| --- | --- | --- | --- | --- | --- | --- | --- |
| Model 2 vs. Model 1 | C-index | T | PFS | 0.0324 | 0.0043~0.0700 | 0.0433 | |
|  |  | V | PFS | 0.0622 | 0.0037~0.1334 | 0.0306 | |
|  | IDI | T | 1-Year PFS | 0.1090 | 0.0160~0.2300 | 0.0070 | |
|  |  |  | 2-Year PFS | 0.0500 | 0.0000~0.1360 | 0.0400 | |
|  |  | V | 1-Year PFS | 0.0640 | -0.0300~0.2870 | 0.3120 | |
|  |  |  | 2-Year PFS | 0.0600 | -0.0110~0.2140 | 0.1330 | |
|  | NRI | T | 1-Year PFS | 0.2690 | 0.0240~0.4950 | 0.0270 | |
|  |  |  | 2-Year PFS | 0.3260 | 0.1010~0.5260 | 0.0070 | |
|  |  | V | 1-Year PFS | 0.3020 | -0.1840~0.6450 | 0.2330 | |
|  |  |  | 2-Year PFS | 0.0890 | -0.1620~0.4600 | 0.2860 | |
|  |  |  |  |  |  |  | |
| Model 3 vs. Model 1 | C-index | T | PFS | 0.1347 | 0.0628~0.2209 | <0.0001 | |
|  |  | V | PFS | 0.0811 | 0.0183~0.1597 | 0.0034 | |
|  | IDI | T | 1-Year PFS | 0.1710 | 0.0670~0.3270 | <0.0001 | |
|  |  |  | 2-Year PFS | 0.2890 | 0.1100~0.4470 | <0.0001 | |
|  |  | V | 1-Year PFS | 0.1120 | -0.0010~0.3360 | 0.0530 | |
|  |  |  | 2-Year PFS | 0.1670 | 0.0480~0.3200 | 0.0070 | |
|  | NRI | T | 1-Year PFS | 0.4020 | 0.1790~0.6620 | <0.0001 | |
|  |  |  | 2-Year PFS | 0.5920 | 0.3580~0.7720 | <0.0001 | |
|  |  | V | 1-Year PFS | 0.5880 | 0.1610~0.7890 | 0.0130 | |
|  |  |  | 2-Year PFS | 0.5260 | 0.1560~0.6980 | 0.0130 | |

1. C-index: Concordance Index; IDI: Integrated Discrimination Improvement; NRI: Net Reclassification Indices;
2. T: Training; V: Validation;
3. CI: Confidence Interval;

### Tab.S7 Result of Random Sample Splitting in Two Modified Datasets

| Predictors | Modified Training Dataset (n=61) | |  | Modified Validation Dataset (n=48) | |
| --- | --- | --- | --- | --- | --- |
|  | HR Mean  (95%CI) | C-index Mean（95%CI） |  | HR Mean  (95%CI) | C-index Mean（95%CI） |
| TNM stage | 5.8747  (3.0645~10.6197) | 0.8265  (0.7821~0.8768) |  | 6.2131  (2.5767~12.7002) | 0.8279  (0.7706~0.8862) |
| Operation History | 2.8201  (1.3964~5.1032) |  |  | 2.9456  (1.215~6.5420) |  |
| sIL-2R | 1.2906  (1.1441~1.4779) |  |  | 1.2990  (1.0986~1.5307) |  |
| CA724 | 1.4299  (1.0682~1.7336) |  |  | 1.3983  (0.9189~1.8352) |  |

HR: Hazard Ratio;

CI: Confidence Interval;

C-index: concordance index;

### Tab.S8 TRIPOD Checklist: Prediction Model Development and Validation (https://www.tripod-statement.org/resources/)

| Section/Topic | Item |  | Checklist Item | **Page** |
| --- | --- | --- | --- | --- |
| Title and abstract | | | | |
| Title | 1 | D;V | Identify the study as developing and/or validating a multivariable prediction model, the target population, and the outcome to be predicted. | 1 |
| Abstract | 2 | D;V | Provide a summary of objectives, study design, setting, participants, sample size, predictors, outcome, statistical analysis, results, and conclusions. | 1-4 |
| Introduction | | | | |
| Background and objectives | 3a | D;V | Explain the medical context (including whether diagnostic or prognostic) and rationale for developing or validating the multivariable prediction model, including references to existing models. | 4-6 |
|  | 3b | D;V | Specify the objectives, including whether the study describes the development or validation of the model or both. | 7 |
| Methods | | | | |
| Source of data | 4a | D;V | Describe the study design or source of data (e.g., randomized trial, cohort, or registry data), separately for the development and validation data sets, if applicable. | 8 |
|  | 4b | D;V | Specify the key study dates, including start of accrual; end of accrual; and, if applicable, end of follow-up. | 8 |
| Participants | 5a | D;V | Specify key elements of the study setting (e.g., primary care, secondary care, general population) including number and location of centres. | 8 |
|  | 5b | D;V | Describe eligibility criteria for participants. | 9 |
|  | 5c | D;V | Give details of treatments received, if relevant. | 10 |
| Outcome | 6a | D;V | Clearly define the outcome that is predicted by the prediction model, including how and when assessed. | 10-11 |
|  | 6b | D;V | Report any actions to blind assessment of the outcome to be predicted. | 11 |
| Predictors | 7a | D;V | Clearly define all predictors used in developing or validating the multivariable prediction model, including how and when they were measured. | 8,10,13 |
|  | 7b | D;V | Report any actions to blind assessment of predictors for the outcome and other predictors. | 11 |
| Sample size | 8 | D;V | Explain how the study size was arrived at. | 13 |
| Missing data | 9 | D;V | Describe how missing data were handled (e.g., complete-case analysis, single imputation, multiple imputation) with details of any imputation method. | 13 |
| Statistical analysis methods | 10a | D | Describe how predictors were handled in the analyses. | 14-15 |
|  | 10b | D | Specify type of model, all model-building procedures (including any predictor selection), and method for internal validation. | 15-16 |
|  | 10c | V | For validation, describe how the predictions were calculated. | 17 |
|  | 10d | D;V | Specify all measures used to assess model performance and, if relevant, to compare multiple models. | 12 |
|  | 10e | V | Describe any model updating (e.g., recalibration) arising from the validation, if done. | 12 |
| Risk groups | 11 | D;V | Provide details on how risk groups were created, if done. | none |
| Development vs. validation | 12 | V | For validation, identify any differences from the development data in setting, eligibility criteria, outcome, and predictors. | 12,15,16 |
| Results | | | | |
| Participants | 13a | D;V | Describe the flow of participants through the study, including the number of participants with and without the outcome and, if applicable, a summary of the follow-up time. A diagram may be helpful. | 13 |
|  | 13b | D;V | Describe the characteristics of the participants (basic demographics, clinical features, available predictors), including the number of participants with missing data for predictors and outcome. | 13-14 |
|  | 13c | V | For validation, show a comparison with the development data of the distribution of important variables (demographics, predictors and outcome). | 13 |
| Model development | 14a | D | Specify the number of participants and outcome events in each analysis. | 14-15 |
|  | 14b | D | If done, report the unadjusted association between each candidate predictor and outcome. | 14-15 |
| Model specification | 15a | D | Present the full prediction model to allow predictions for individuals (i.e., all regression coefficients, and model intercept or baseline survival at a given time point). | 15-16 |
|  | 15b | D | Explain how to the use the prediction model. | 15,18,19 |
| Model performance | 16 | D;V | Report performance measures (with CIs) for the prediction model. | 16-17 |
| Model-updating | 17 | V | If done, report the results from any model updating (i.e., model specification, model performance). | none |
| Discussion | | | | |
| Limitations | 18 | D;V | Discuss any limitations of the study (such as nonrepresentative sample, few events per predictor, missing data). | 20 |
| Interpretation | 19a | V | For validation, discuss the results with reference to performance in the development data, and any other validation data. | none |
|  | 19b | D;V | Give an overall interpretation of the results, considering objectives, limitations, results from similar studies, and other relevant evidence. | 19-21 |
| Implications | 20 | D;V | Discuss the potential clinical use of the model and implications for future research. | 21-22 |
| Other information | | | | |
| Supplementary information | 21 | D;V | Provide information about the availability of supplementary resources, such as study protocol, Web calculator, and data sets. | Supplementary data |
| Funding | 22 | D;V | Give the source of funding and the role of the funders for the present study. | 23 |

## Supplementary Figures: Fig. S1 to Fig.S7

### Fig.S1. Flow Chart of Data Screening

a. Deficiency of both Qi and Yin according to Identification of Syndrome Based on TCM Theories;

b. ECOG: Eastern Cooperative Oncology Group;

### Fig.S2 Heat Map of Correlation between Bio-Features and Clinical Characteristics for Preliminarily Selecting Prognostic Variables of LASSO-Cox Regression

“CC” (correlation of coefficient) is a pairwise statistic derived from the following formula: where r represents the spearman’s correlation coefficient.

| A | 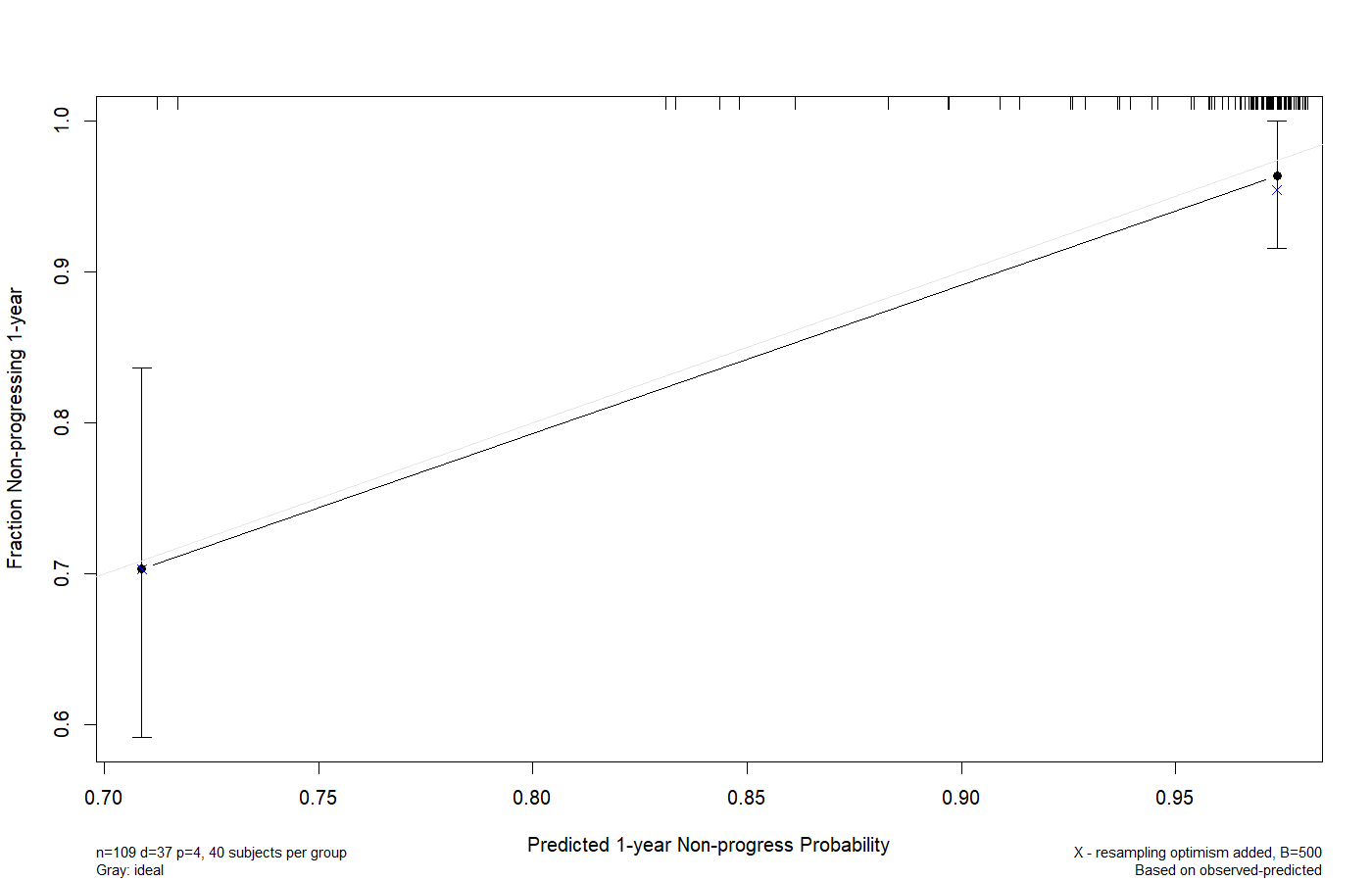 | 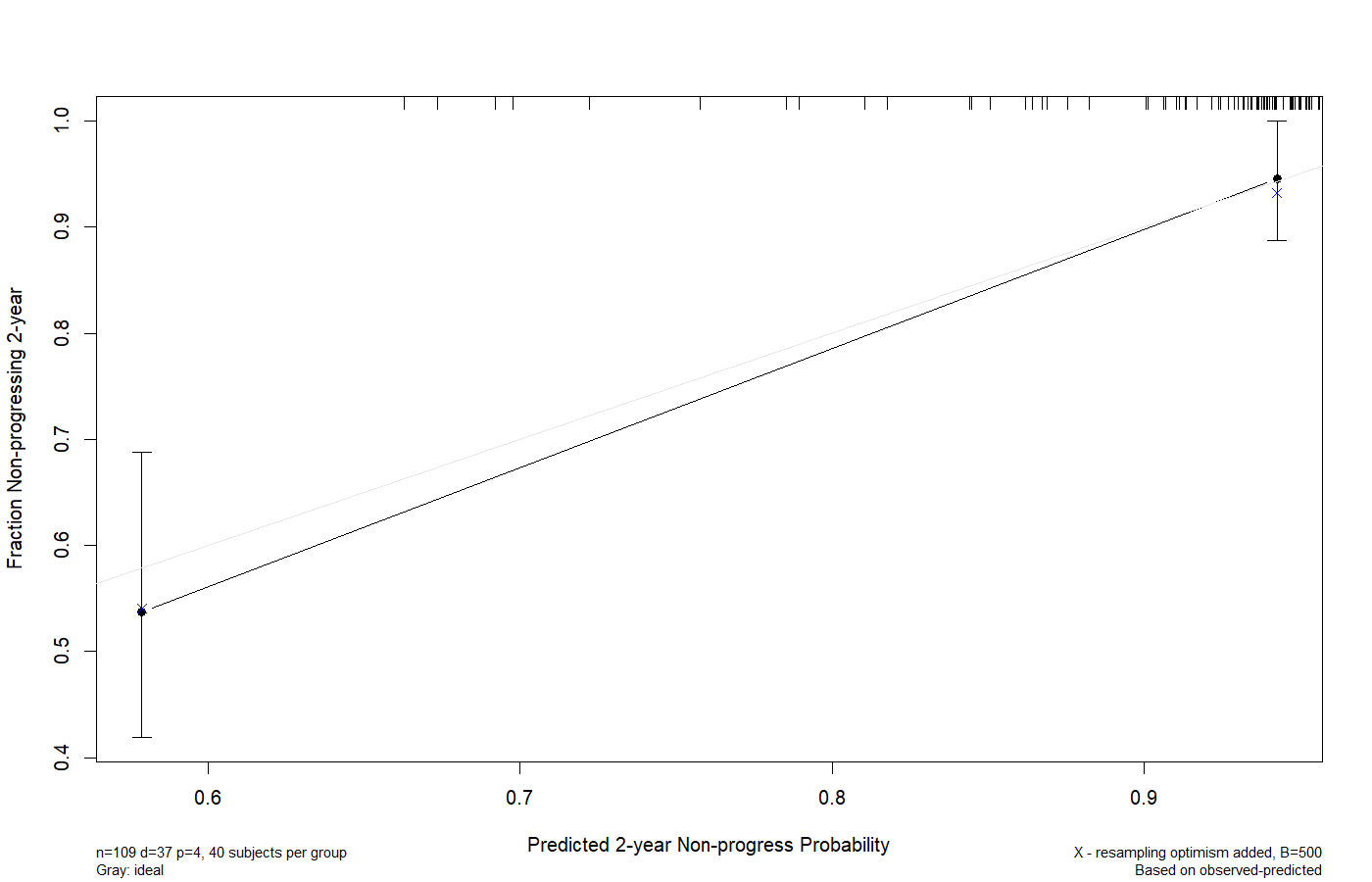 |
| --- | --- | --- |
| B | 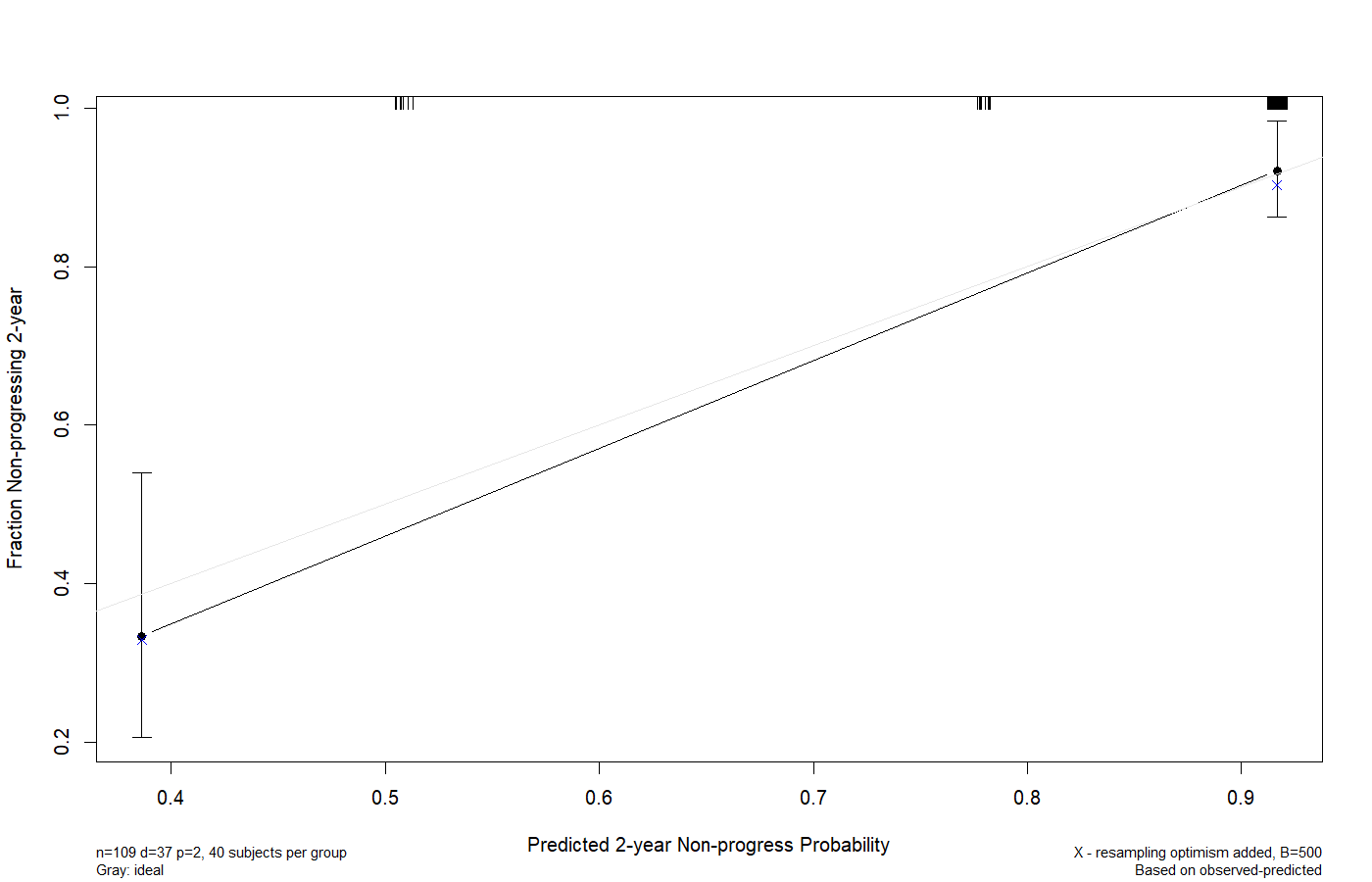 | 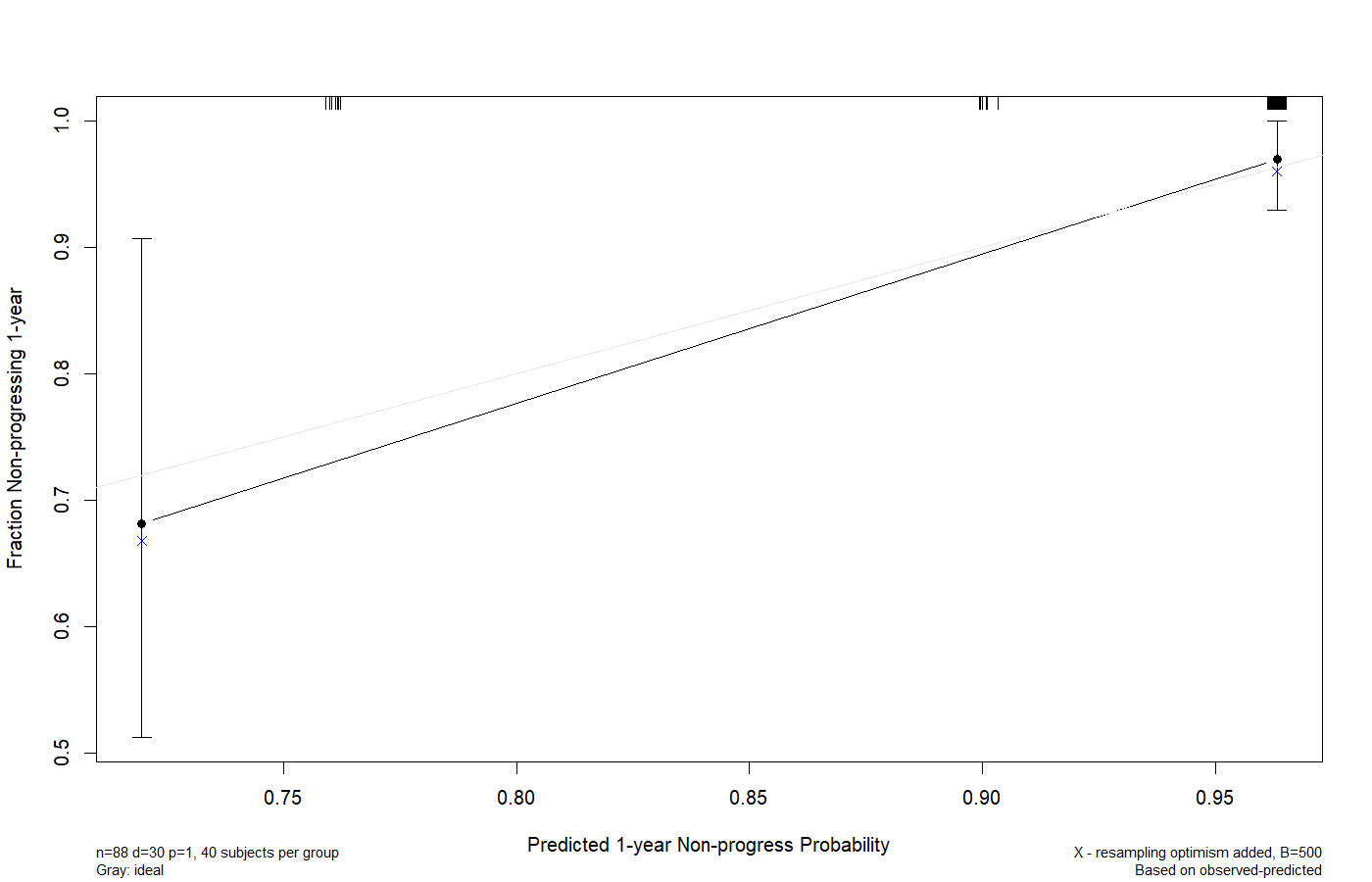 |
| C | 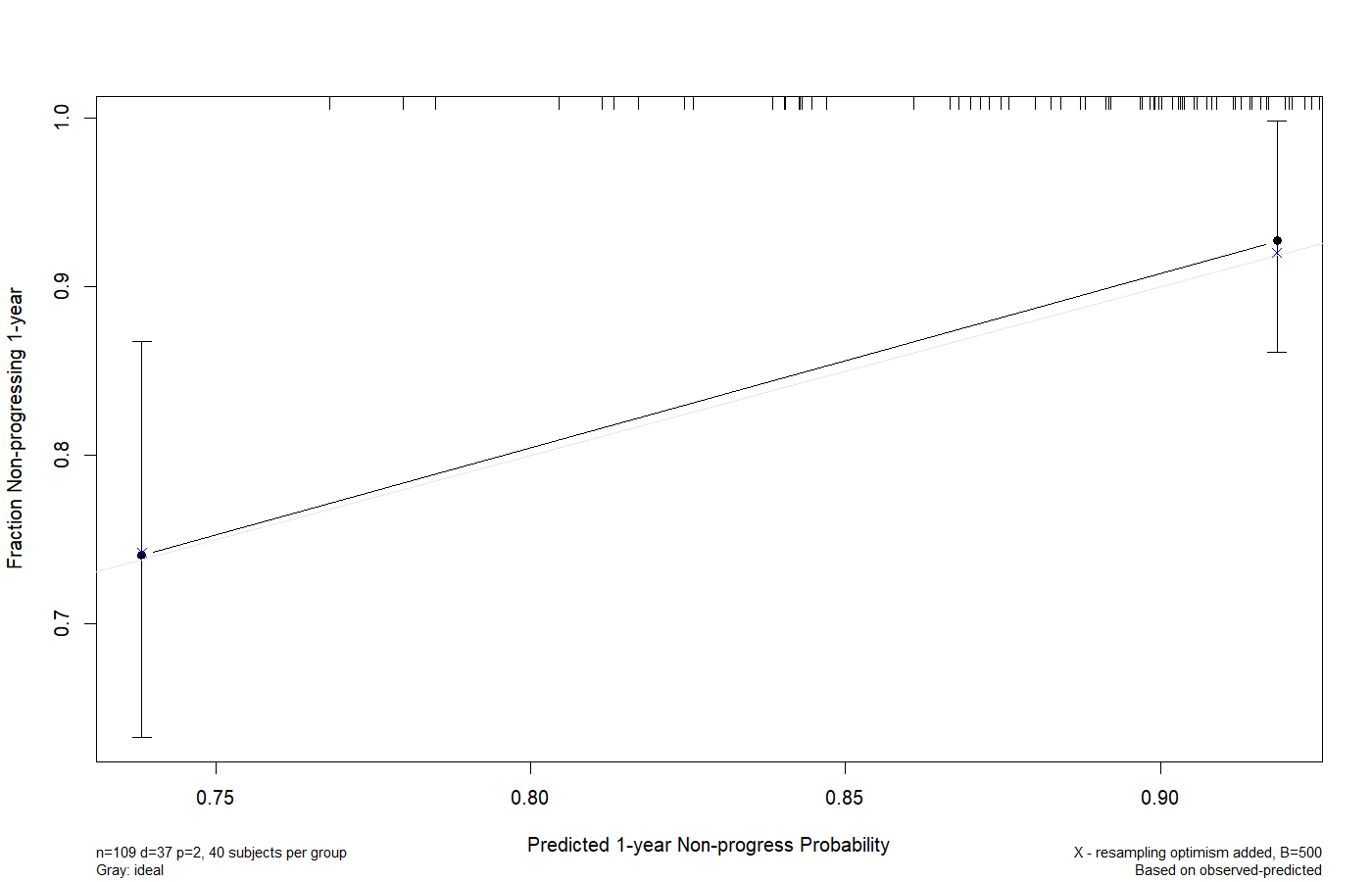 | 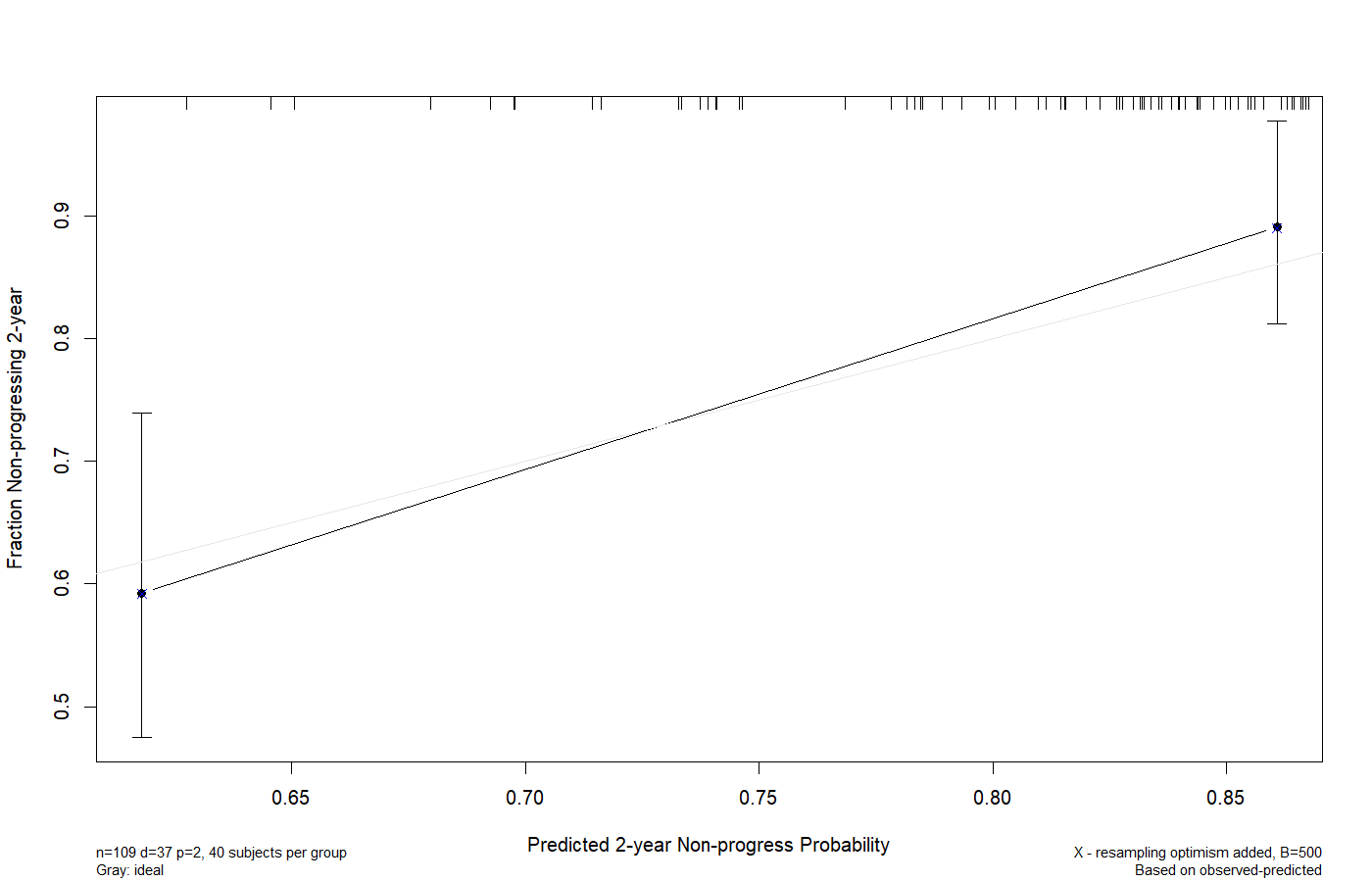 |

### Fig.S3 Calibration of Training Dataset (n=109).

### A. Calibration of 1-year and 2-year PFS in Model 1; B. Calibration of 1-year and 2-year PFS in Model 2; C. Calibration of 1-year and 2-year PFS in Model 3;

| A | 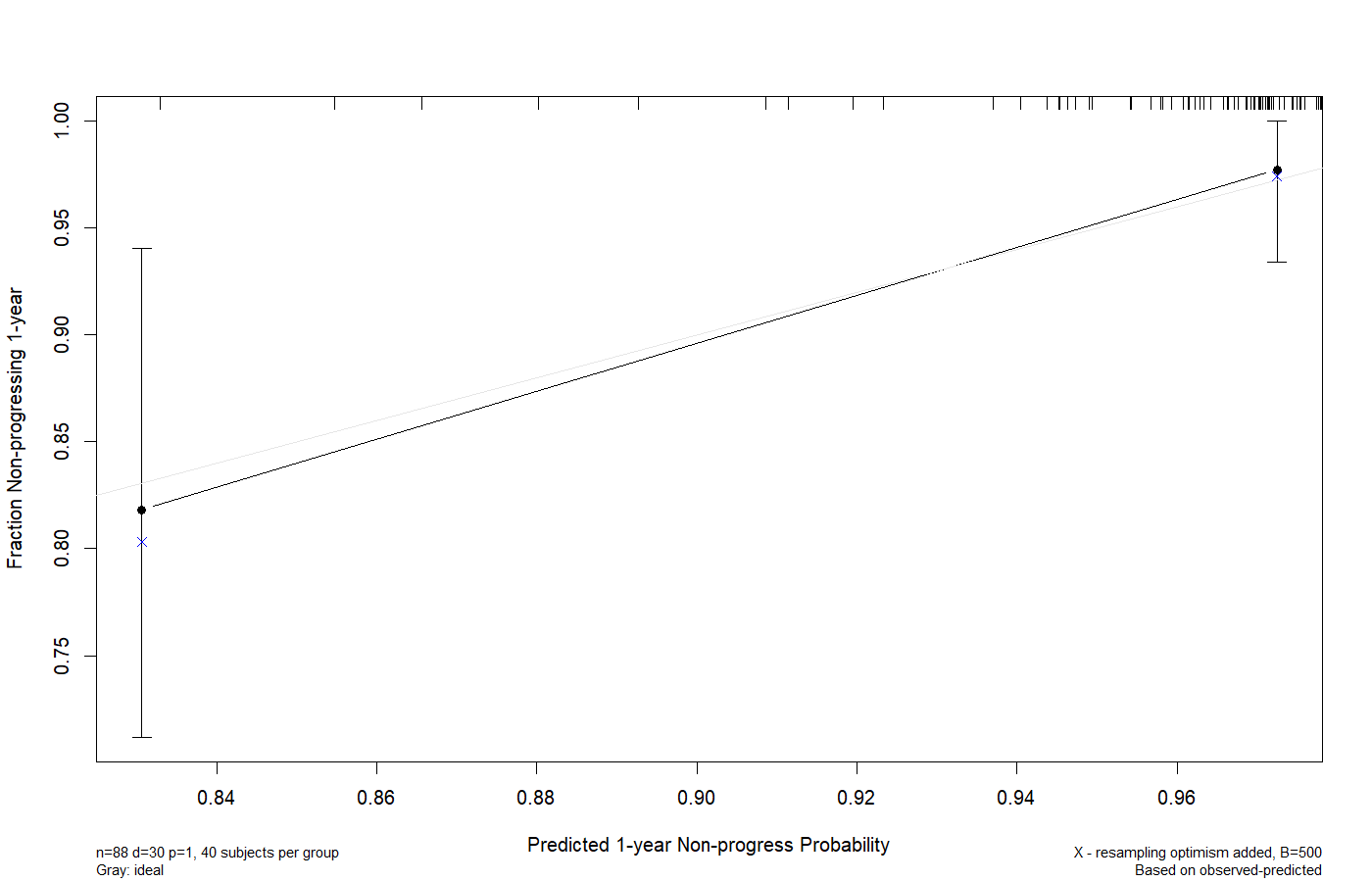 | 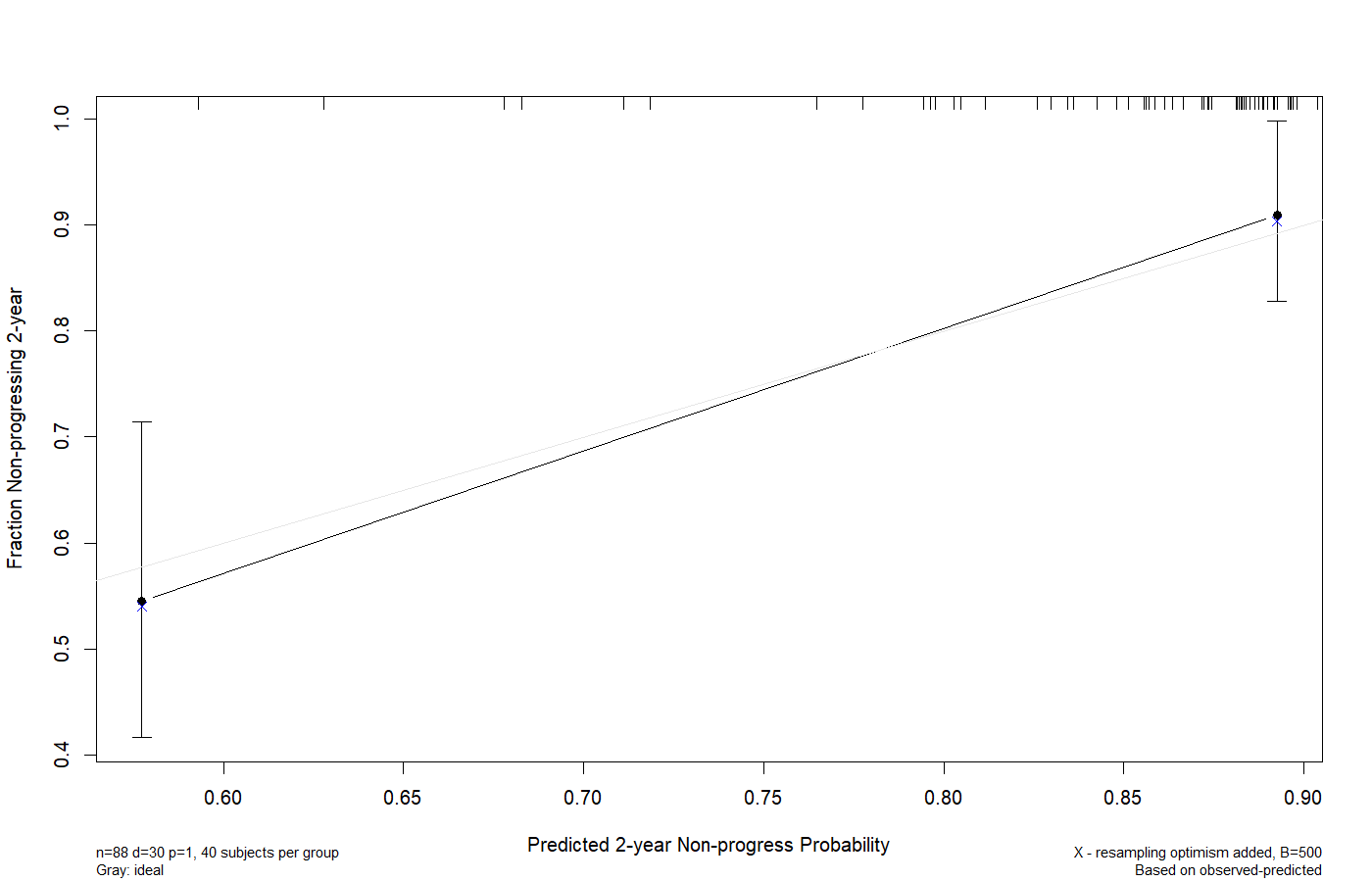 |
| --- | --- | --- |
| B | 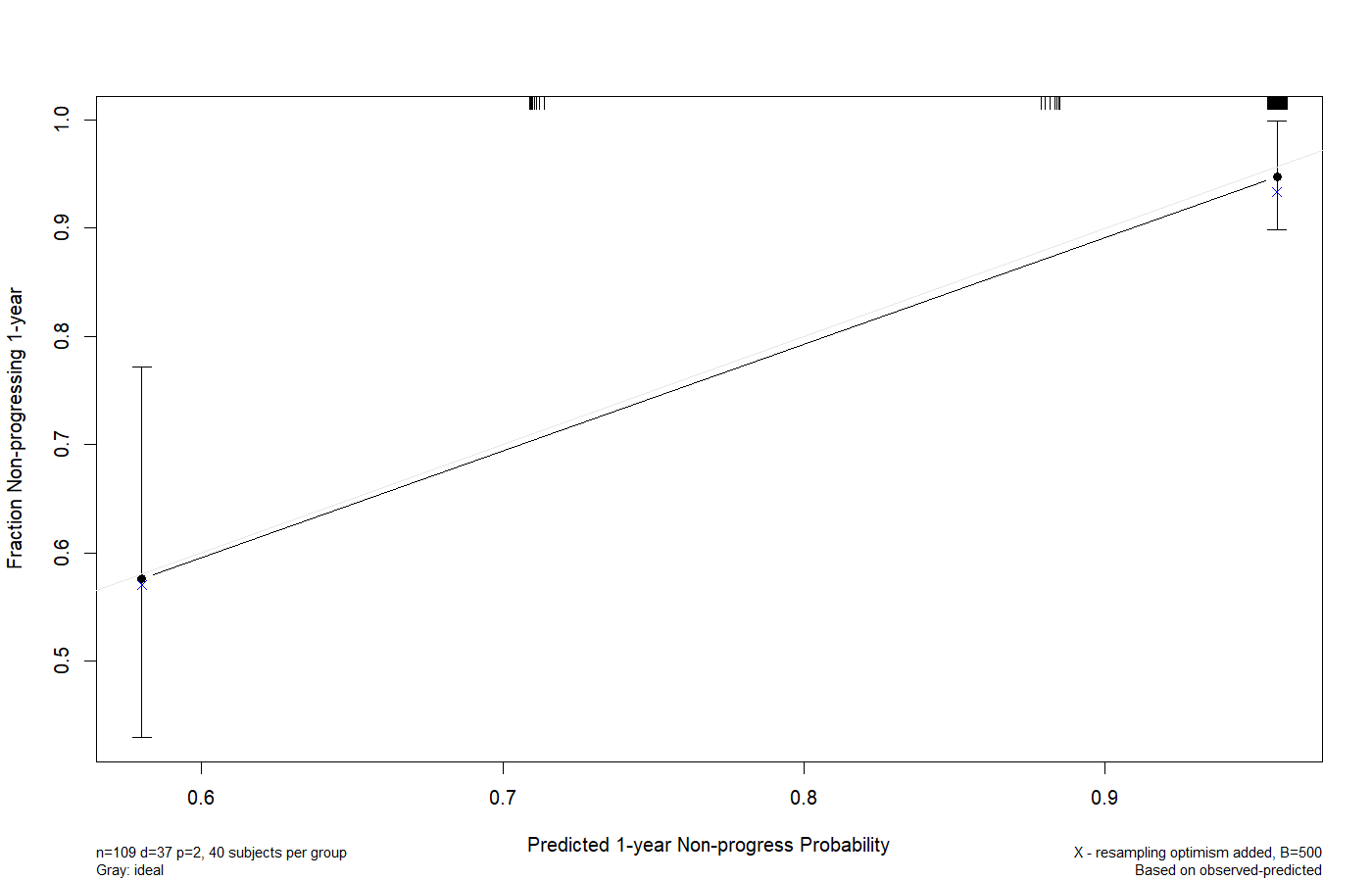 | 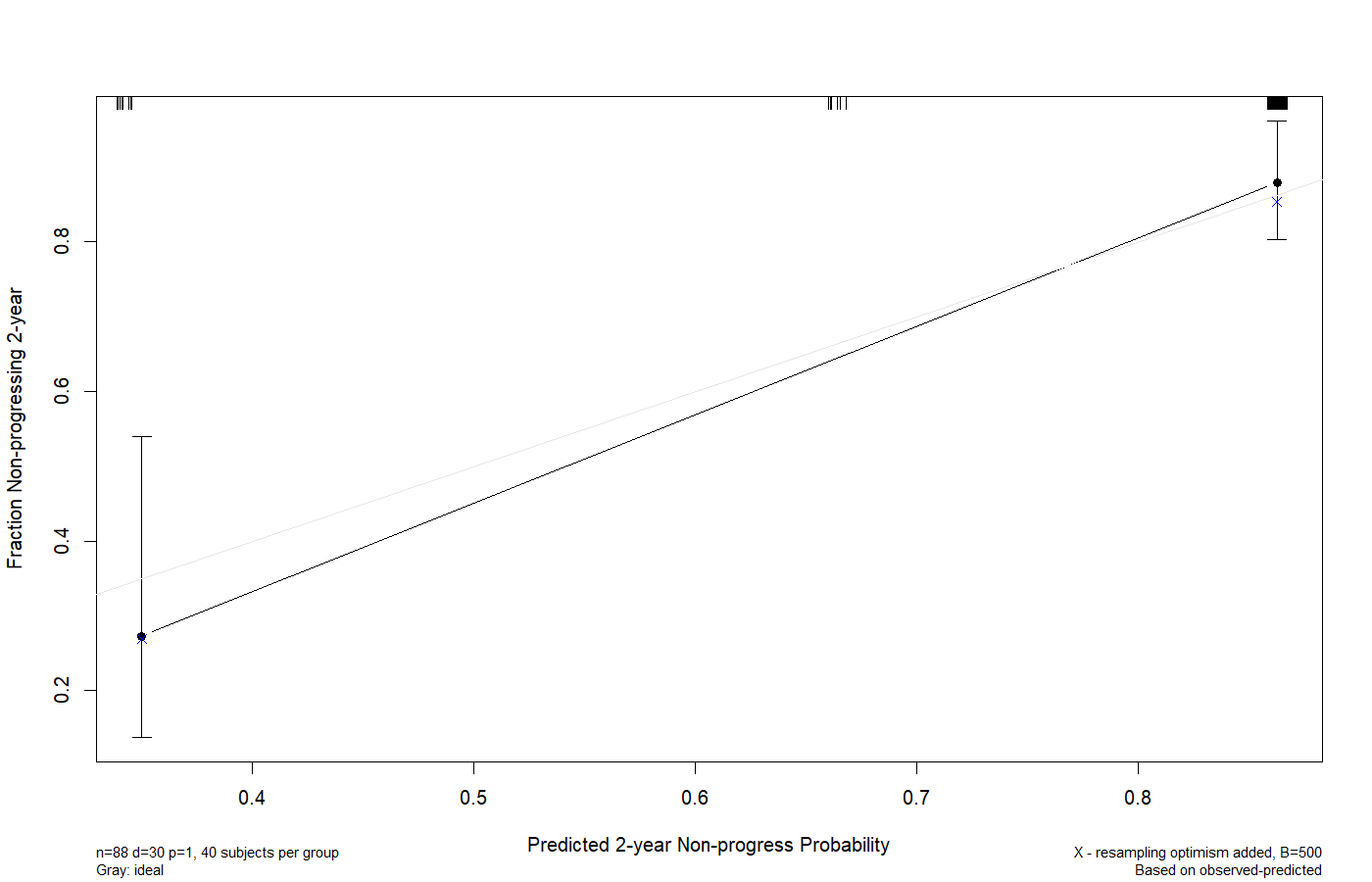 |
| C | 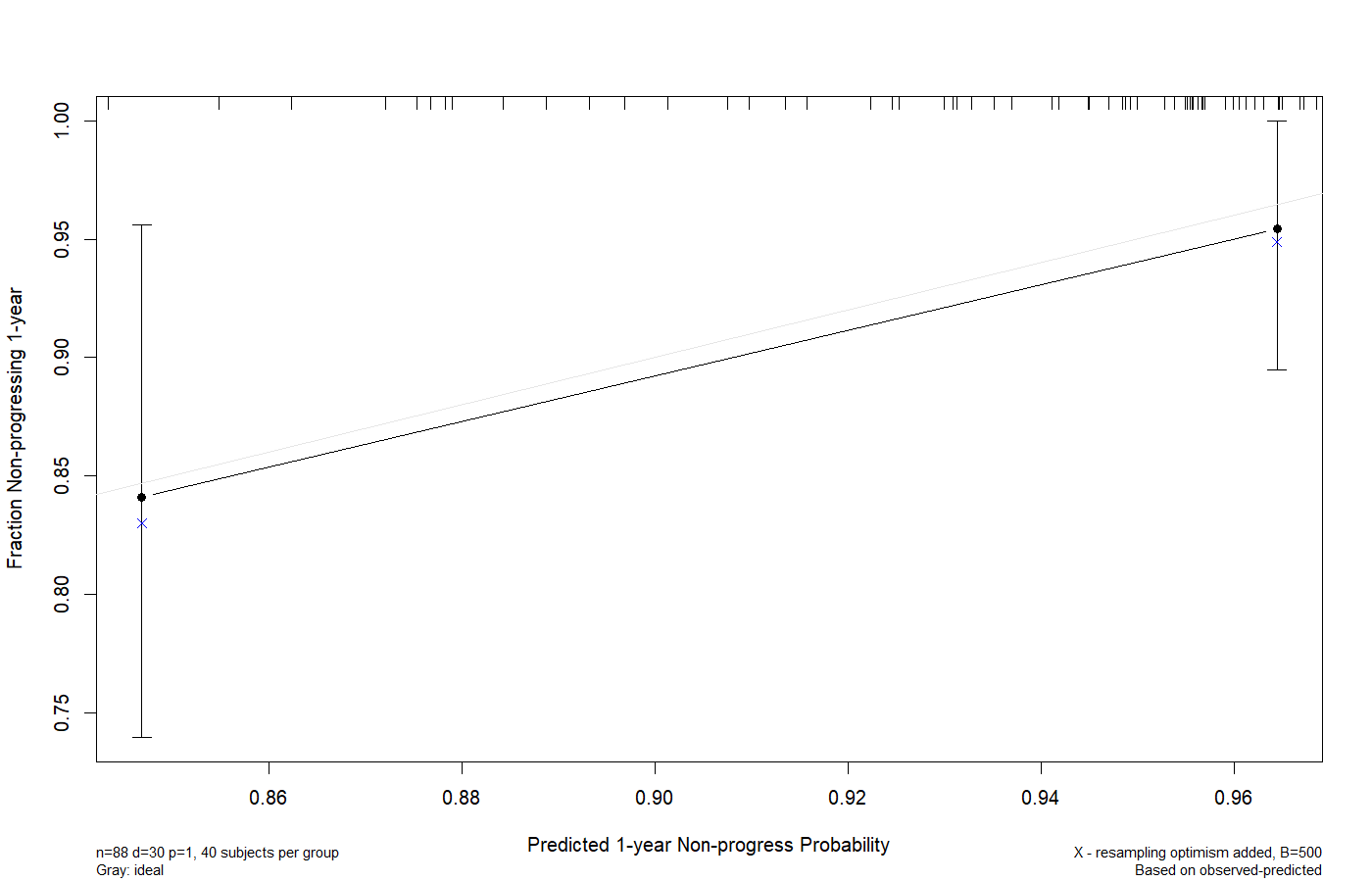 | 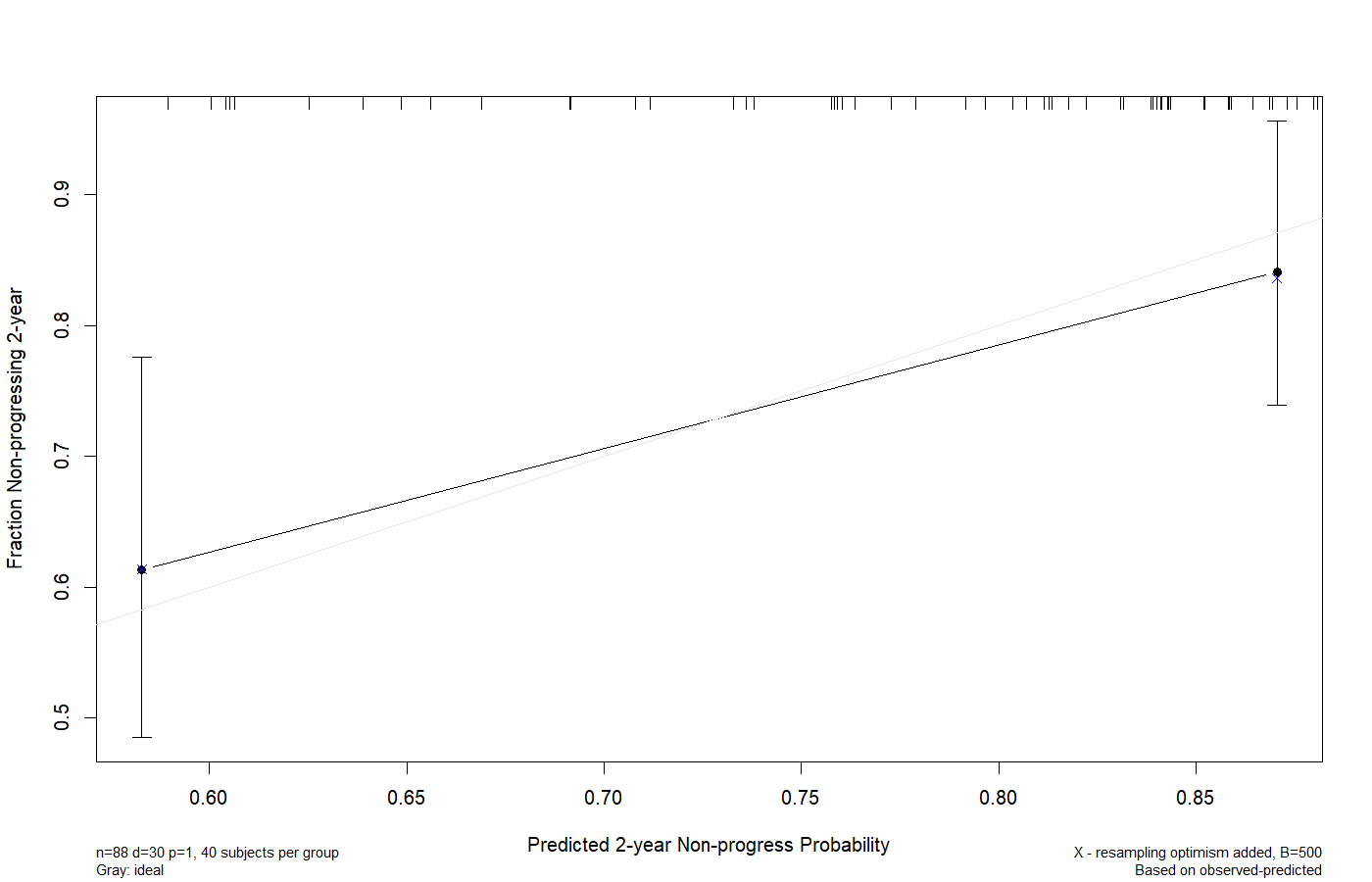 |

### Fig.S4 Calibration of Validation Dataset (n=88)

### A. Calibration of 1-year and 2-year PFS in Model 1; B. Calibration of 1-year and 2-year PFS in Model 2; C. Calibration of 1-year and 2-year PFS in Model 3;

| A  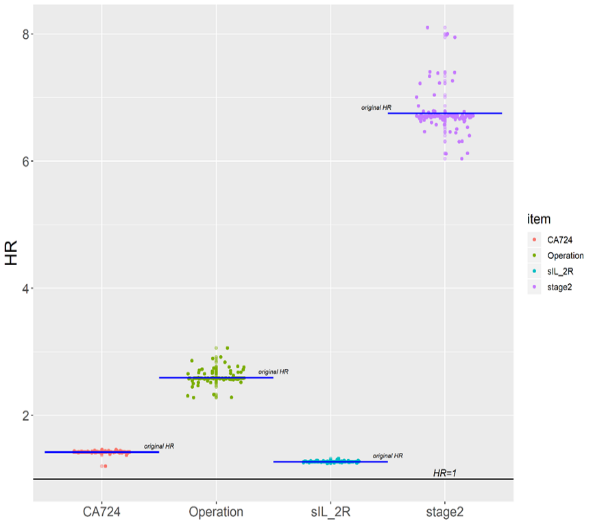 | B  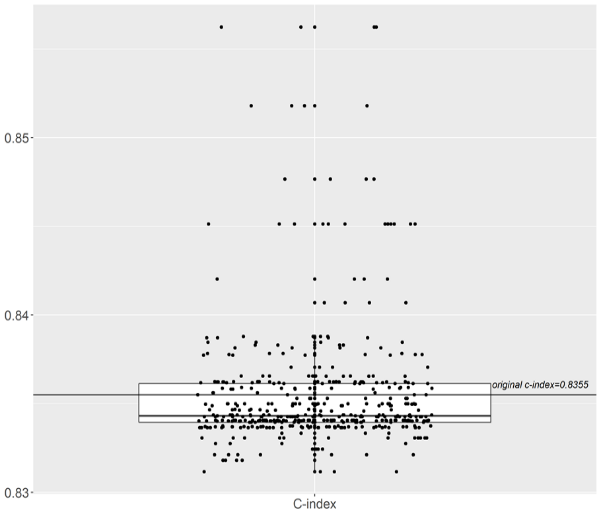 |
| --- | --- |
| C  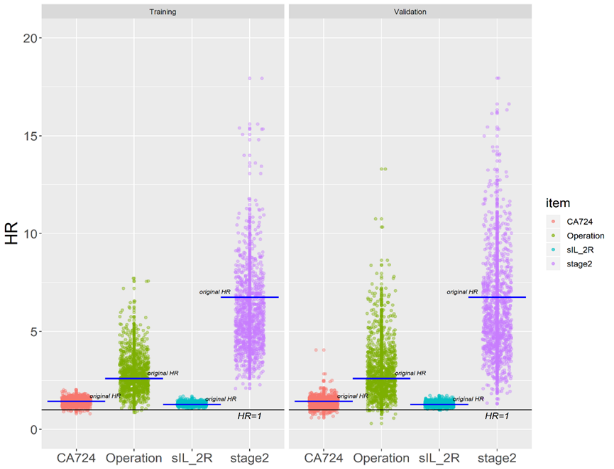 | D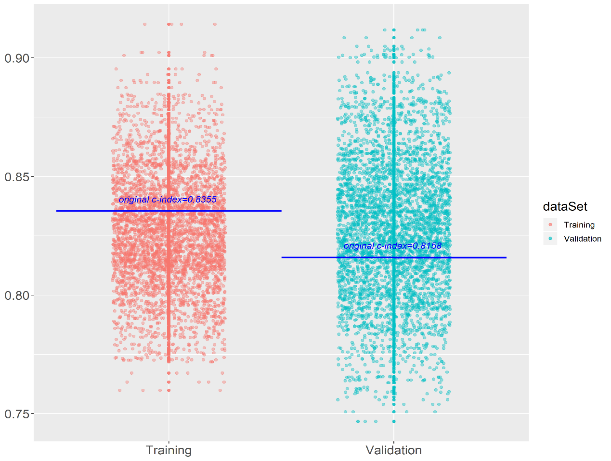 |

### Fig.S5 Result of Sensitivity Analysis Based on Training Dataset(n=88) A. Distribution of HR Values for 4 Predictors; B. Average C-index for 4 Predictors; C. Distribution of HR values for Both Modified Datasets; D. C-index for Both Modified Dataset

HR: Hazard Ratio


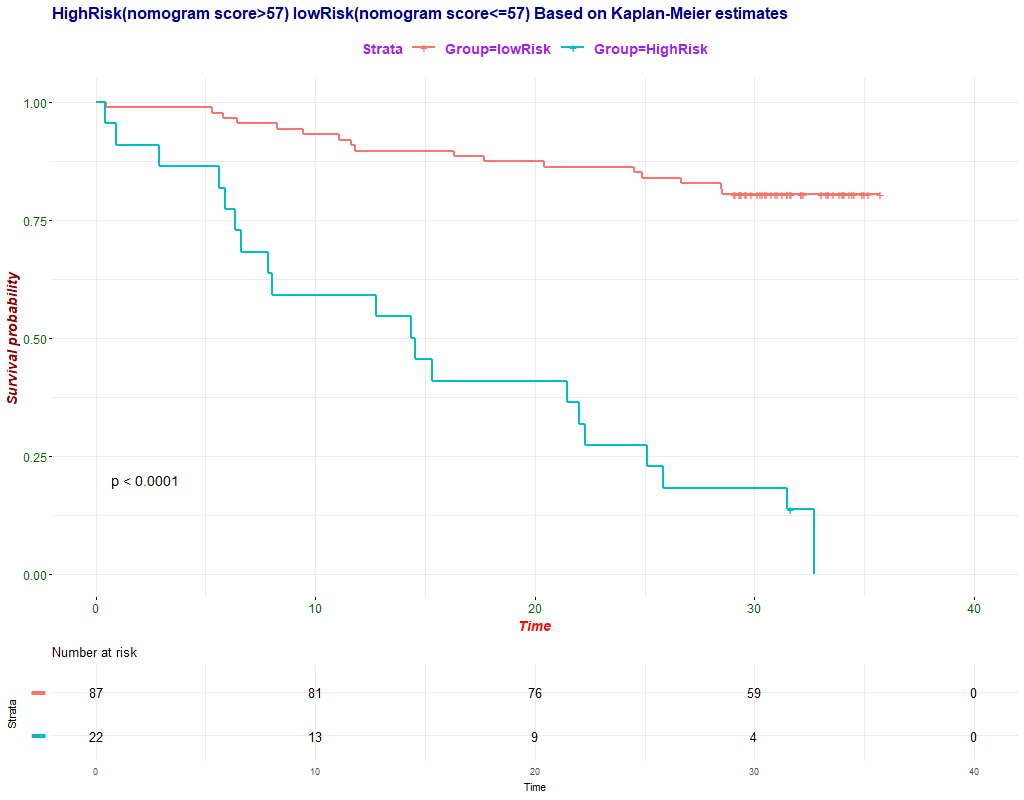


### Fig.S6 Survival Probability Estimated by Kaplan-Meier Curve of High-risk Group and Low-risk Group in Training Dataset (n=109)


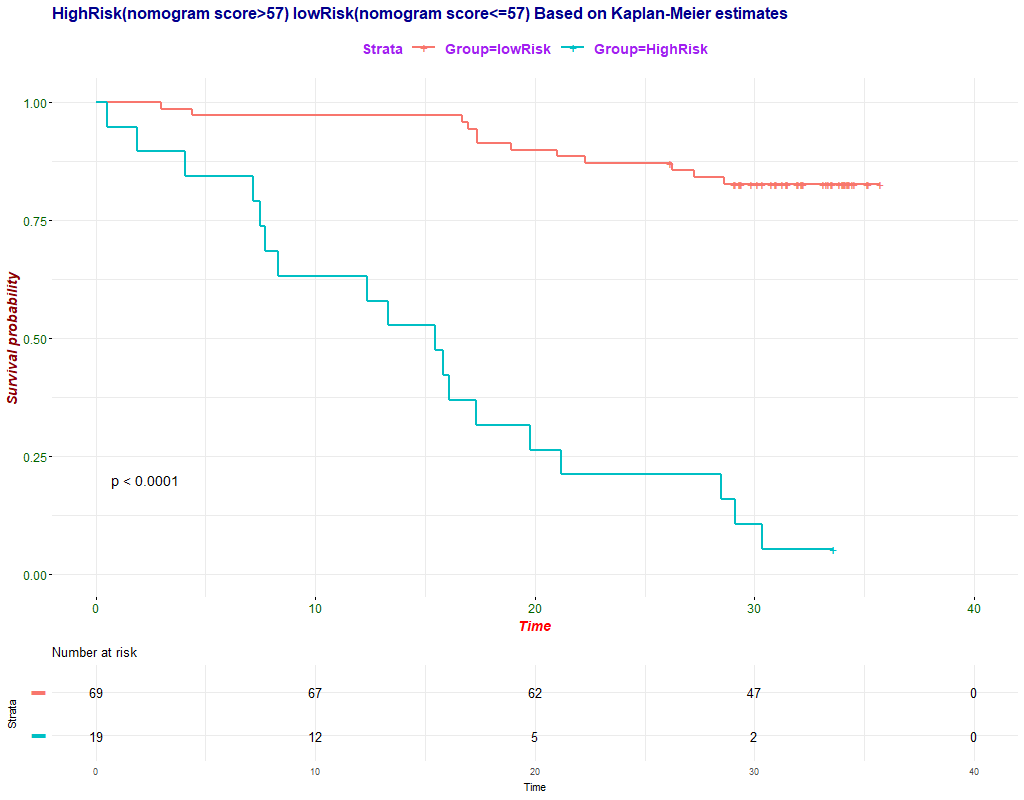


### Fig.S7 Survival Probability Estimated by Kaplan-Meier Curve of High-risk Group and Low-risk Group in Validation Dataset (n=88)
